# The efferocytosis-related genes of SLC26A6, TYRO3, and PDK4 have been identified as predictors of prognosis in hepatocellular carcinoma and are associated with the immune status

**DOI:** 10.7150/ijms.120781

**Published:** 2026-03-04

**Authors:** Jiao-Jiao Yang, Qing Ouyang, Xin-Xin Zeng, Wen-Jian Cen, Ling Deng, Gao-Ping Song, Fang Wang, Li-Yue Sun

**Affiliations:** 1 Department of Oncology, The Seventh Affiliated Hospital, Sun Yat-Sen University, Shenzhen, Guangdong, China.; 2 Department of Hepatobiliary, General Hospital of Southern Theatre Command of PLA, Guangzhou, China.; 3 Second Department of Oncology, The Affiliated Guangdong Second Provincial General Hospital of Jinan University, Guangzhou, China.; 4 Department of Molecular Diagnostics, Sun Yat-sen University Cancer Center, Guangzhou, Guangdong, China.; 5 Department of Health Management Centre, Zhongshan Hospital, Fudan University, China.; 6 Department of General Practice, Zhongshan Hospital, Fudan University, China.; 7 Department of Health Management Centre, The First Affiliated Hospital of Jinan University, Guangzhou, China.

**Keywords:** hepatocellular carcinoma, efferocytosis, prognosis, nomogram, immune

## Abstract

**Background:**

Efferocytosis plays a critical role in clearing apoptotic tumor cells and suppressing inflammation in hepatocellular carcinoma (HCC). This study aimed to identify efferocytosis-related genes (ERGs) with prognostic value and develop a predictive model for HCC outcomes.

**Methods:**

Using public HCC transcriptomic and clinical data, we identified 13 differentially expressed ERGs (DE-ERGs) from 3,866 DEGs and 74 known ERGs. Cox regression analysis selected SLC26A6, TYRO3, and PDK4 as key prognostic genes for risk model construction. The model, combined with pathologic T stage in a nomogram, showed high predictive accuracy for patient survival.

**Results:**

Totally 13 DE-ERGs were gained by overlapping 3,866 DEGs and 74 ERGs, and SLC26A6, TYRO3, and PDK4 were identified as prognosis genes for constructing a risk model with highly proficient in assessing the risk of HCC patients. Then, both risk score and pathologic T stage were recognized as independent factors prognosticating the outcome of HCC patients. Afterwards, we constructed a nomogram utilizing risk score and pathologic T stage to achieve high accuracy in predicting the survival outcomes of HCC patients. Groups at low risk demonstrated enrichment in pathways related to biometabolism and immune response, such as "fatty acid metabolism" and "complement and coagulation cascades". Additionally, the strongest positive and negative correlation were observed from activated CD4^+^T cell and TYRO3 (cor = 0.37), as well as natural killer cell and SLC26A6 (cor = -0.35), respectively. And risk score exhibited strong predictive capacity in response to immunotherapy. Moreover, lncRNA-miRNA-mRNA network included complex interaction pairs, such as TYRO3-hsa-miR-203b-5p-NUTM2A-AS1. There were 61 drugs with significant differences in IC_50_ between the high and low risk groups, such as BI.2536 and PD-173074. Single-cell analysis identified hepatocytes as the key cell population, exhibiting dynamic prognostic gene expression during differentiation and disease-specific alterations in cell-cell communication through ligand-receptor interactions.

**Conclusion:**

We identified three prognostic genes associated with efferocytosis in HCC and integrated them into a risk prognostic model. These genes not only serve as signatures for predicting HCC prognosis but also offer insights into the treatment of HCC.

## 1. Introduction

Currently, liver cancer is the sixth most common cancer worldwide and the second leading cause of cancer-related deaths^1^. Hepatocellular carcinoma (HCC) is the most common type of primary liver cancer, originating in the hepatocytes, the predominant cell type in the liver. HCC represents a significant global health challenge, particularly in regions with high rates of chronic liver disease^1^. The development of HCC is closely linked to chronic liver diseases such as hepatitis B and C virus infections, alcoholic liver disease, and non-alcoholic fatty liver disease (NAFLD). Other risk factors include aflatoxin exposure, diabetes, obesity, and genetic predispositions, chronic inflammation and fibrosis resulting from these conditions can lead to the malignant transformation of hepatocytes, ultimately giving rise to HCC^2^. HCC exhibits significant regional differences worldwide, with higher prevalence generally observed in developing countries. This is primarily due to the high infection rates of chronic hepatitis B and C in these regions. Conversely, in developed countries, HCC is more commonly associated with liver cirrhosis, which is often a consequence of chronic alcohol abuse and chronic hepatitis C^3^. Early-stage HCC may not present noticeable symptoms, leading to over 60% of HCC patients being diagnosed at an advanced stage^4^. Unfortunately, for those with advanced HCC, the 5-year survival rate remains low, typically ranging from 5% to 15%, depending on factors such as the patient's overall health, the stage of the cancer at diagnosis, and the available treatment options^5^. Despite some HCC patients undergoing curative liver resection, high rates of postoperative recurrence and metastasis remain major challenges to survival for HCC patients^6^. Thus, it is imperative to explore specific and novel prognostic and diagnostic biomarkers for HCC. By analyzing the molecular mechanisms of these genes and constructing prognostic risk models, we can assess the prognostic risk of patients. These genetic characteristics have significant clinical value in predicting the progression of HCC and treatment responses, thereby providing new targets for the development of novel drugs and immunotherapies.

The term "efferocytosis" refers to the process where one cell engulfs another, typically involving macrophages engulfing apoptotic cells. This intricate process is critical for maintaining tissue homeostasis and preventing inflammation^7^. i. By rapidly clearing apoptotic cells, efferocytosis helps prevent the release of cellular debris and toxic intracellular substances, thereby reducing the occurrence of inflammatory responses^7^. ii. Efferocytosis modulates the activity of immune cells and antigen presentation, aiding in the maintenance of immune homeostasis^8^. iii. Efferocytosis also plays a role in the development and progression of various diseases, including autoimmune disorders, infections, cancer, and cardiovascular diseases^8^-^10^. iv. Efferocytosis can exert positive or negative effects on the body through various signaling pathways and the release of cytokines^9^, ^11^. Cancer is a disease characterized by high rates of cellular apoptosis^12^. In this context, efferocytosis may play a crucial role by efficiently clearing and processing apoptotic tumor cells in HCC and suppressing inflammatory responses^13^. This process is vital for maintaining liver tissue homeostasis and preventing inflammation^14^. Recent studies have indicated that dysfunction in the efferocytosis process in HCC can lead to impaired clearance of apoptotic cells, which in turn may promote tumor growth and metastasis^14^. This dysfunction in clearing apoptotic cells efficiently can contribute to a pro-tumorigenic environment by preventing the normal resolution of inflammation and potentially enhancing the tumor's ability to evade the immune response^15^. Therefore, further research into the function and mechanisms of efferocytosis in HCC is crucial for developing new treatment strategies and improving the prognosis for HCC patients.

This study identified prognostic genes related to efferocytosis in HCC through a series of bioinformatics approaches, utilizing transcriptome datasets from the University of California Santa Cruz (UCSC) Xena, the International Cancer Genome Consortium (ICGC), and the Gene Expression Omnibus (GEO) databases. We constructed a risk model based on prognostic genes and explored their potential mechanisms of action. Specifically, we developed an lncRNA-miRNA-mRNA network centered on these prognostic genes, performed GSEA enrichment analysis, and analyzed immune infiltration, immunotherapy response, and drug sensitivity in high- and low-risk groups. These findings provide new insights for the clinical diagnosis, prevention, and treatment of HCC.

## 2. Materials and Methods

### 2.1 Data source

Expression matrix and clinical information of The Cancer Genome Atlas (TCGA)-Liver hepatocellular carcinoma (LIHC) from University of California Santa Cruz (UCSC) xena database (https://xenabrowser.net/datapages/) were downloaded. The dataset contained 424 tumour tissue samples with available survival information for 363 HCC and 50 normal samples. HCC data set (GSE14520) and single cell RNA sequencing data set (GSE149614), from Gene Expression Omnibus (GEO) database (https://www.ncbi.nlm.nih.gov/gds). This dataset included liver tissue samples from 225 HCC (221 HCC samples with survival information were retained) and 220 control patients based on GPL3921 platform^16^. The dataset GSE149614 contains transcriptomic profiles of hepatocellular carcinoma (HCC) and adjacent normal liver tissues. And we obtained the transcriptomic and survival information for Liver Cancer - RIKEN, JP (LIRI-JP) dataset, which included 232 HCC samples, from International Cancer Genome Consortium (ICGC) database (https://dcc.icgc.org/). Moreover, a total of 74 efferocytosis-related genes (ERGs) were retrieved from previous publications^15^, ^17^. All the data and clinical information in this study were retrieved from public databases. Therefore, ethics committee approval and written informed consent were not necessary.

### 2.2 Identification of differentially expressed genes (DEGs) and differentially expressed ERGs (DE-ERGs)

DEGs were screened out from HCC and normal samples [|log_2_Fold Change (FC)| > 1 and adj.*P* < 0.05] by DESeq2 package (v 1.36.0)^18^ within TCGA-LIHC dataset. Volcano plot and heatmap were plotted to visualize DEGs through ggplot2 package (v 3.3.6)^19^ and heatmap package (v 1.0.12)^20^, respectively. Subsequently, we acquired DE-ERGs by overlapping DEGs and 74 ERGs via ggvenn package (v 1.2.2)^21^, the 74 cell efferocytosis genes are shown in **Table S1**.

### 2.3 The expression, chromosome location, and enrichment analysis of DE-ERGs

In an effort to demonstrate the distribution of DE-ERGs expression between HCC and normal samples in TCGA-LIHC dataset, box plot, volcano plot and heatmap of DE-ERGs expression were plotted, respectively. And the chromosomal distribution of DE-ERGs was visualized using RCircos package (v 1.2.2)^22^. For the purpose of understanding the potential biological functions and signaling pathways associated with DE-ERGs, Gene ontology (GO) and Kyoto encyclopedia of genes and genomes (KEGG) enrichment analysis of DE-ERGs were completed via ClusterProfiler package (v 4.4.4)^23^ (*P* < 0.05). The results were visualized by enrichplot package (v 1.16.2)^24^.

### 2.4 Identification and expression analysis of prognosis genes

So as to screen out prognosis genes, in the first place, we conducted univariate Cox regression analysis on DE-ERGs within TCGA-LIHC dataset [*P* < 0.05 and Hazard Ratio (HR) ≠ 1], and then we carried out multivariate Cox regression analysis to further refine the selection of prognosis genes (step function, *P* < 0.2). The expression profiles of prognosis genes were depicted using box plot, while the trends in expression were verified in GSE14520 dataset. In addition, we stratified HCC patients from TCGA-LIHC dataset into high and low expression groups according to median expression values of prognosis genes. Subsequently, we used survival package (v 3.4-0)^25^ to generate Kaplan-Meier (K-M) survival curves and assess differences in survival among distinct expression groups. This allowed us to investigate the impact of the expression of prognosis genes on the prognosis of HCC patients.

### 2.5 Construction and validation of risk model

In TCGA-LIHC dataset, risk score for each HCC patient was calculated based on relative expression of prognosis genes and their corresponding multivariate Cox coefficient. The formula used to calculate the risk scores was as follows: 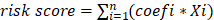, where Xi represented relative expression of prognosis genes i, coefi denoted multivariate Cox coefficient associated with prognosis genes i. Next, HCC patients were stratified into high and low risk groups via median value of the risk score. Subsequently, the distinct risk groups underwent K-M survival analysis to evaluate differences in survival. And receiver operating characteristic (ROC) curves were plotted via survival ROC package (v 1.0.3)^26^ to determine area under curve (AUC) at 1, 2 and 3 years, providing further assessment of the validity of the risk model. Additionally, we presented the distribution of risk scores, survival status of HCC patients, and the expression levels of prognosis-related gene between distinct risk groups. We further validated the risk model in GSE14520 and LIRI-JP datasets.

### 2.6 Construction of independent prognostic model

Initially, univariate Cox regression analysis was performed to combine risk scores and clinical characteristics (age, gender, stage, histologic grade, and pathologic TNM stage) of HCC patients (*P* < 0.05 and HR ≠ 1) in TCGA-LIHC dataset. Afterwards, independent prognostic factors were determined through multivariate Cox regression analysis (*P* < 0.05 and HR ≠ 1) via the results obtained from univariate Cox regression. To conduct a more comprehensive analysis of the survival of HCC patients, we utilized independent prognostic factors obtained and plotted a nomogram via rms package (v 6.3-0)^27^. The predictive performance of nomogram was assessed using calibration curves.

### 2.7 Assessing the relationship between risk scores and clinical characteristics

With the intention of exploring the relationship between risk scores and clinical characteristics (age, gender, stage, histologic grade, and pathologic TNM stage), we compared the differences in clinical characteristics based on risk scores among different subgroups via TCGA-LIHC dataset. Subsequently, differences among subgroups defined by each clinical characteristic were studied by Wilcoxon test (P < 0.05).

### 2.8 Gene Set Enrichment Analysis (GSEA) of distinct risk groups

In order to gain insights into pathways associated with distinct risk groups, differential expression analysis was initially conducted on distinct risk groups using DESeq2 package within TCGA-LIHC dataset and ranked according to log_2_FC. Thereafter, GSEA was performed based on rank results using ClusterProfiler package. The background gene set utilized for this analysis was c2.cp.kegg.v7.0.symbols.gmt, obtained from GSEA database (http://www.gsea-MSigdb.org/gsea/msigdb) (P<0.05, FDR <0.25). The top 5 pathways with KEGG enrichment results, ranked in descending order based on their absolute *P* values, were presented. To further verify the applicability of KEGG, we used Hallmark in MSigDB for enrichment pathway validation.

### 2.9 Immune infiltration analysis

To examine the variations in immune cell infiltration among distinct risk groups within TCGA-LIHC dataset, we conducted a separate analysis of immune cell infiltration for each HCC sample. This was achieved using single sample gene set enrichment analysis (ssGSEA) algorithm from Gene Set Variation Analysis (GSVA) package (v 1.44.5)^28^. The immune scores for 28 immune cell^29^ were calculated for all samples and visualized using heatmap created with pheatmap package, as well as differences in immune cell proportions from distinct risk groups were compared (*P* < 0.05). With the aim of further investigating the relationship between prognosis genes and differential immune cells, we conducted Spearman correlation calculations and visualized the results via ggcorrplot package (v 0.1.4)^24^.

### 2.10 Analysis of the correlation between risk scores and immunotherapy

Immune checkpoints played a critical role in anti-tumor immunotherapy. In this study, we analyzed the expression levels of 39 immune checkpoints^30^ within TCGA-LIHC dataset and compared the expression differences among distinct risk groups (*P* < 0.05). The correlationship between prognostic genes with differential immune checkpoints was then done by cor function (|correlation (cor)| > 0.3 and p < 0.05). To evaluate the response to immunotherapy in HCC patients within TCGA-LIHC dataset, we utilized Tumor Immune Dysfunction and Exclusion (TIDE) database (http://tide.dfci.harvard.edu/) to calculate the response of these patients. The study visualized the proportion of HCC patients who responded and did not respond to immunotherapy within distinct risk groups, and compared the difference in response to immunotherapy between both groups (*P* < 0.05). Next, HCC patients were classified into two groups: TRUE and FALSE, and difference in risk scores between the two groups was calculated (*P* < 0.05). And ROC curves were plotted using pROC package (v 1.18.5)^31^ to determine the effectiveness of risk scores in predicting the response to immunotherapy.

### 2.11 Gene mutation analysis

In order to accurately demonstrate the mutations of previously identified prognosis genes, we utilized cBioPortal database (https://www.cbioportal.org/) to analyze the mutations of these genes in the TCGA-LIHC dataset. Meanwhile, we investigated the association between the copy number variation (CNV) of prognosis genes and the level of immune cell infiltration using Tumour ImmunoEstimation Resource (TIMER) database (http://cistrome.dfci.harvard.edu/TIMER/). The HCC patients were grouped based on the median value of immune infiltration level, and the association was evaluated with the survival rate of HCC patients.

### 2.12 Construction of lncRNA-miRNA-mRNA network and drug sensitivity analysis

In order to deepen investigate the molecular regulatory mechanisms of prognosis genes in HCC, the miRWalk database (http://mirwalk.umm.uni-heidelberg.de/) was utilized to predict upstream miRNAs of prognosis genes, with a screening criteria of binding > 0.9, accessibility < 0.01, and energy < -18. The starBase database (https://rnasysu.com/encori/) was used to predict the lncRNAs interacting with these upstream miRNAs with the filtering condition of clipExpNum > 5. The lncRNA-miRNA-mRNA relationship network was mapped using Cytoscape software (v 3.8.2)^32^.

Besides, we retrieved drug sensitivity data^33^ from CellMiner database (https://discover.nci.nih.gov/cellminer/home.do). The drugs that underwent "clinical trial validation and FDA approval" screening were subjected to Spearman analysis to assess their correlation with prognosis genes (|cor| > 0.3 and *P* < 0.05), and the top 5 correlations were then visualized.

For further evaluation the sensitivity of HCC patients within TCGA-LIHC dataset to conventional chemotherapeutic drugs, we utilized Genomics of Drug Sensitivity in Cancer (GDSC) database (https://www.cancerrxgene.org/) to predict half maxima inhibitory concentration (IC_50_) of 138 common chemotherapeutic drugs through pRRophetic package (v 0.5)^34^, and compared the IC_50_ values of each drug between distinct risk groups (*P* < 0.05).

### 2.13 The expression of target genes in HCC and normal liver tissue

Paired HCC and adjacent normal liver tissues from 25 patients were obtained from postoperative pathological specimens, followed by DNA extraction and qPCR analysis for target gene expression profiling. The reaction mixture in each well consisted of 2 µl of tissue DNA at a concentration of 5 ng/µl, 5 µl of the mix, and 0.64 µl of primers and probes. The PCR conditions were set to one cycle of 5 minutes at 95°C, followed by 20 cycles of 15 seconds at 95°C and 30 seconds at 64°C, with fluorescence detection occurring at 60°C for 10 seconds over 40 cycles. The relative expression of target gene was calculated with the 2^-△△ct^ method. The relevant PCR primers are shown in **Table S2**. This study was conducted in accordance with the ethical principles of the Declaration of Helsinki and approved by the Ethics Committee of Guangdong Second Provincial General Hospital (Approval No: 2021-KZ-138-03). All human tissue samples, including HCC and adjacent normal liver tissues, were obtained from postoperative pathological specimens with prior written informed consent from each participating patient.

### 2.14 scRNA-seq analysis

To analyze the cellular heterogeneity in the microenvironment of HCC, in the GSE149614 dataset, the "Seurat" package (v 5.0.1)^35^ was utilized for quality control (QC) to filter out cells with exceeded 10% of mitochondrial genes, cells with nCount_RNA under 200 and surpassed 6,000 genes, and cells with nFeature_RNA > 200^35^. Then, in light of the GSE149614 dataset, data were normalized by the "NormalizeData" function in the "Seurat" package (v 5.0.1)^35^, and highly variable genes (HVGs) were selected by the "FindVariableFeatures" function. Next, the "ScaleData" function in the "Seurat" package (v 5.0.1)^35^ was applied to scale data before principal components analysis (PCA). Subsequently, the "JackStraw" function within the "Seurat" package (v 5.0.1)^35^ was applied to execute PCA on HVGs. The "ElbowPlot" function within the "Seurat" package (v 5.0.1)^35^ was thereafter applied to draw a scree plot of the top 30 principal components (PCs), aiming to identify PCs that notably contributed to variation for subsequent analysis (p < 0.05). Afterward, cell cluster analysis was conducted on cells after dimensionality reduction utilizing "FindNeighbors" and "FindClusters" functions (resolution = 0.1, dimension = 30). After that, the cells were clustered by the uniform tSNE clustering method. Annotate the cells based on the references^36^. Finally, through singleR^37^, "HumanPrimaryCellAtlasData", "BlueprintEncodeData", and "ImmuneCellExpressionData" were utilized as reference data for auxiliary annotation, and in accordance with CellMarker database (http://xteam.xbio.top/CellMarker/), marker genes of cell clusters were searched for and cell - type annotation was undertaken.

Differences in proportions between HCC and controls were then compared using chi-square tests, corrected for multiple testing using the Benjamini-Hochberg (p < 0.05) method, and difference-in-difference cell analyses were performed using the “ggplot2” package (v 3.5.1)^38^ for subsequent studies. Additionally, the "ReactomeGSA" package (v 1.16.1)^39^ was leveraged to implement functional enrichment analysis on differential cells and calculate pathway expression. In order to identify the key cells, based on the GSE149614 dataset, the expression of key genes in the differential cells was detected, and the cells with the highest expression level of key genes were selected as the key cells. Subsequently, the differentiation trajectory of hepatocytes was detected through the "monocle" package (v 2.30.1)^40^, and the density curve was plotted using the "ggpubr" package (v 0.4.0) (https://cran.r-project.org/web/packages/ggpubr/index.html) to reveal the developmental stages of different cell clusters and the dynamic expression of prognostic genes at different differentiation stages of key cells.

Furthermore, cell-cell communication among key cells and other cells was examined via the "CellChat" package (v 1.6.1)^41^, and a ligand-receptor bubble plot of cell-cell communication was produced.

### 2.15 Statistical analysis

All analyses were executed in R language (v 4.2.2). Differences between groups were analyzed by Wilcoxon test. *P* < 0.05 was considered statistically significant.

## 3. Results

### 3.1 A sum of 13 DE-ERGs were screened out

**Figure 1:**
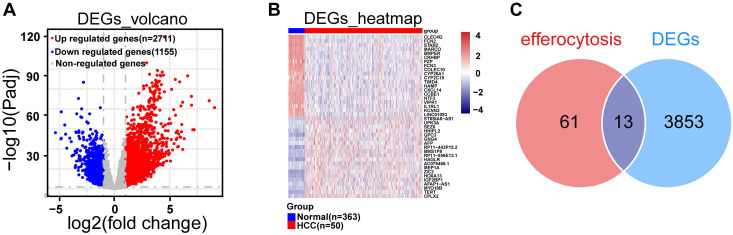
Differential expression analysis. **A.** Volcano plot of differential genes in the TCGA-LIHC dataset, blue dots represent down-regulated differentially expressed genes, red dots represent up-regulated differentially expressed genes, and gray are genes that are not statistically significant, with each dot representing one gene. **B.** The heatmap illustrated the expression patterns of the top 20 up-regulated DEGs and top 20 down-regulated DEGs within the TCGA-LIHC dataset. **C.** Venn diagram for differential efferocytosis factor.

**Figure 2:**
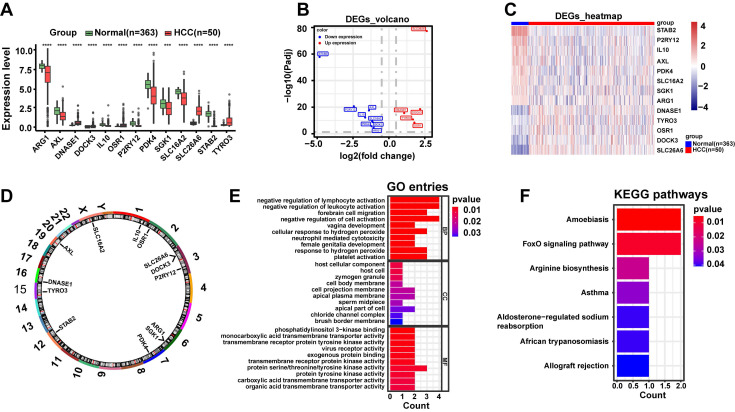
DE-ERGs were linked to immune functions and disease signaling pathways. **A.** Box plot of DE-ERGs expression, red represents the disease group and green represents the normal group. **B.** Volcano plot of DE-ERGs expression, blue represents low expression, red represents high expression. **C.** Heat map of DE-ERGs expression, red represents the disease group, blue represents the normal group. **D.** Differential cytokine chromosome circle diagram. **E.** The horizontal coordinate count represents the number of genes under the GO term, the vertical coordinate represents the name of each GO term, and the filled color of the bar represents the level of significance, with darker red indicating higher significance. **F.** Differential efferocytosis factor KEGG enrichment analysis, the vertical coordinate represents the name of each KEGG pathway, and the color of the bar represents the level of significance, with darker red indicating higher significance. Data are presented as mean ± SD of three independent experiments. ***p < .001, ****p < .0001. Statistical analysis was performed using Wilcoxon test.

In TCGA-LIHC dataset, we screened out 3,866 DEGs, including 2,711 up-regulated and 1,155 down-regulated from HCC and normal samples through differential expression analysis (**Fig. 1A**). The heatmap illustrated the expression pattern of the top 20 up-regulated and down-regulated DEGs (**Fig. 1B**). By overlapping DEGs and 74 ERGs, a sum of 13 DE-ERGs were acquired **(Fig. 1C)**, namely ARG1, AXL, DNASE1, DOCK3, IL10, OSR1, P2RY12, PDK4, SGK1, SLC16A2, SLC26A6, STAB2, and TYRO3 **(Fig. 2A)**.

### 3.2 DE-ERGs were associated with immune-related biological functions and disease-related signaling pathways

Subsequently, we exhibited the distribution of DE-ERGs expression from HCC and normal samples in TCGA-LIHC dataset. The results clearly demonstrated that DNASE1, DOCK3, OSR1, SLC26A6, and TYRO3 displayed high expression in HCC sample (*P* < 0.0001), and the opposite was true for ARG1, AXL, IL10, P2RY12, PDK4, SGK1, SLC16A2, and STAB2 (*P* < 0.001) (**Fig. 2A-C**). And these DE-ERGs were distributed across multiple chromosomes with, SLC16A2 located on X chromosome and the remaining 12 DE-ERGs located on autosomes (**Fig. 2D**). Additionally, we performed GO and KEGG enrichment analyses to investigate potential biological roles and signaling pathways of DE-ERGs. Explicitly, DE-ERGs were principally associated with "negative regulation of lymphocyte activation", "negative regulation of leukocyte activation", and "forebrain cell migration" in GO entries (**Fig. 2E**), and were predominantly enriched in "amoebiasis", "FoxO signaling pathway", and "arginine biosynthesis" and other KEGG pathways (**Fig. 2F**). The results revealed that DE-ERGs were not only associated with immune-related biological functions, but also closely related to disease-related signaling pathways.

### 3.3 SLC26A6, TYRO3, and PDK4 were identified as prognosis genes

**Figure 3:**
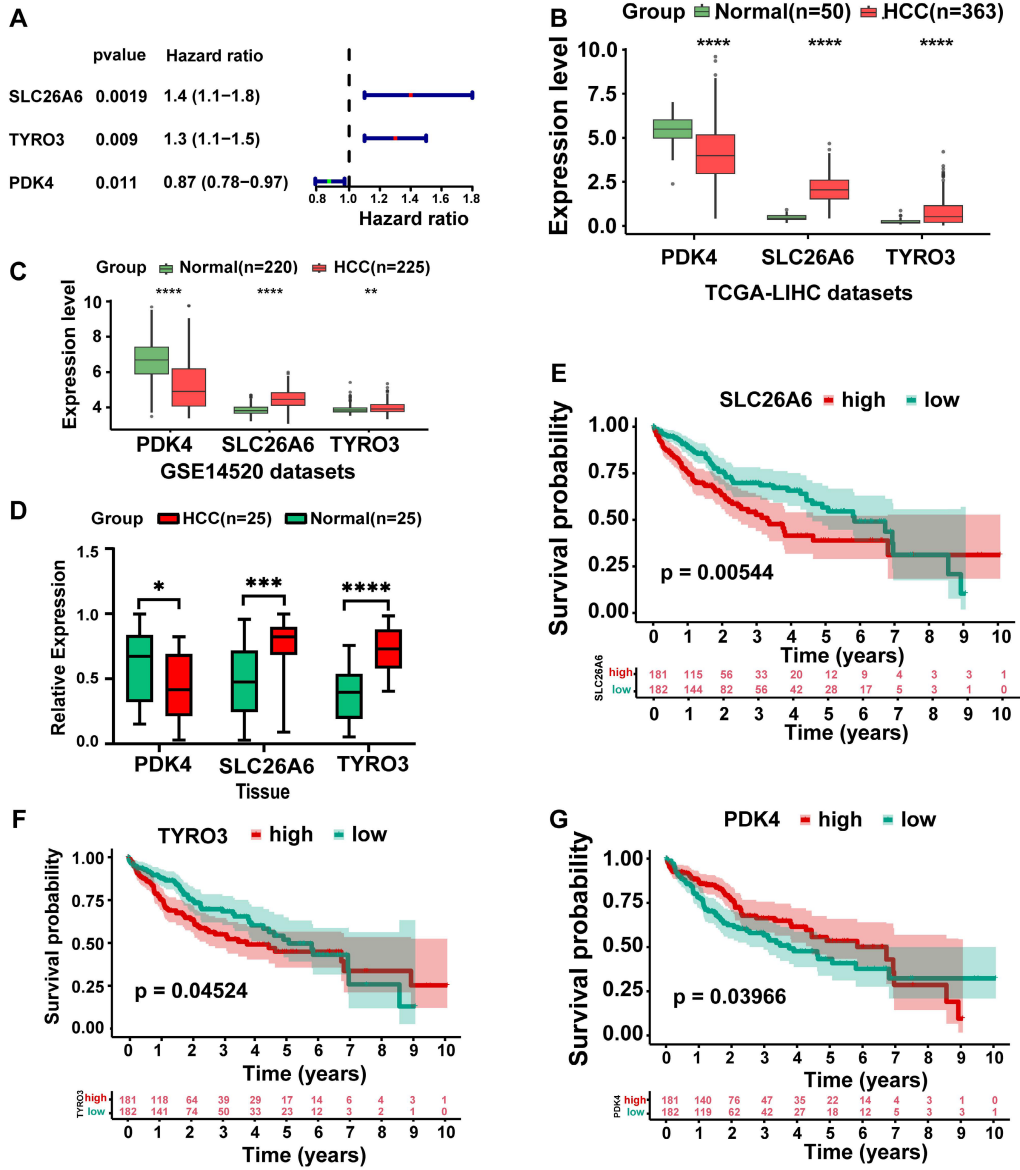
Identification and expression analysis of prognosis-related genes. **A.** Univariate and multivariate Cox regression were conducted on DE-ERGs within the TCGA-LIHC dataset to identify ERGs (P < 0.05). **B.** Box-line plot of HCC prognostic gene expression (TCGA-LIHC). **C.** Box-line plot of HCC prognostic gene expression (GSE14520). **D.** Box-line plot of HCC prognostic gene expression (Tissue). **E.** High and low gene expression KM curves (SLC26A6). **F.** High and low gene expression KM curves (TYRO3). **G.** High and low gene expression KM curves (PDK4). The statistical analysis employed the log-rank test and Wilcoxon test. Data were presented as mean ± SD. *p < .05, **p < .01, ***p < .001, ****p < .0001. KM Kaplan-Meier.

In TCGA-LIHC dataset, we screened out three genes: SLC26A6, TYRO3, and PDK4 through univariate Cox regression analysis (*P* < 0.05) (**Fig. 3A**). The identified genes were subsequently incorporated into a multivariate Cox regression analysis to identify prognosis genes. Ultimately, these genes were deemed significant for prognosis (*P* < 0.2) (**Table 1**). Among the prognosis genes, SLC26A6 and TYRO3 exhibited a positive association with HCC and prognostic risk (HR > 1). Conversely, PDK4 showed a negative association with HCC and prognostic risk (HR < 1). Remarkably, SLC26A6, TYRO3, and PDK4 exhibited consistent and significant expression trends in both TCGA-LIHC and GSE14520 datasets (*P* < 0.01) (**Fig. 3B, 3C**). We subsequently performed q-PCR validation in both HCC tissues and normal tissues, and the results were consistent with the findings from public database analyses (**Fig. 3D**). Specifically, SLC26A6 and TYRO3 displayed high expression levels in HCC samples compared to normal samples (*P* < 0.01), whereas PDK4 demonstrated the opposite trend (*P* < 0.0001). And significant differences in survival rate were observed among prognosis genes from distinct expression groups (*P* < 0.05) (**Fig. 3E-G**). These findings provided further validation for the accuracy of our results in identifying prognosis genes.

### 3.4 Risk model exhibited strong proficiency in assessing the risk of HCC patients

**Figure 4:**
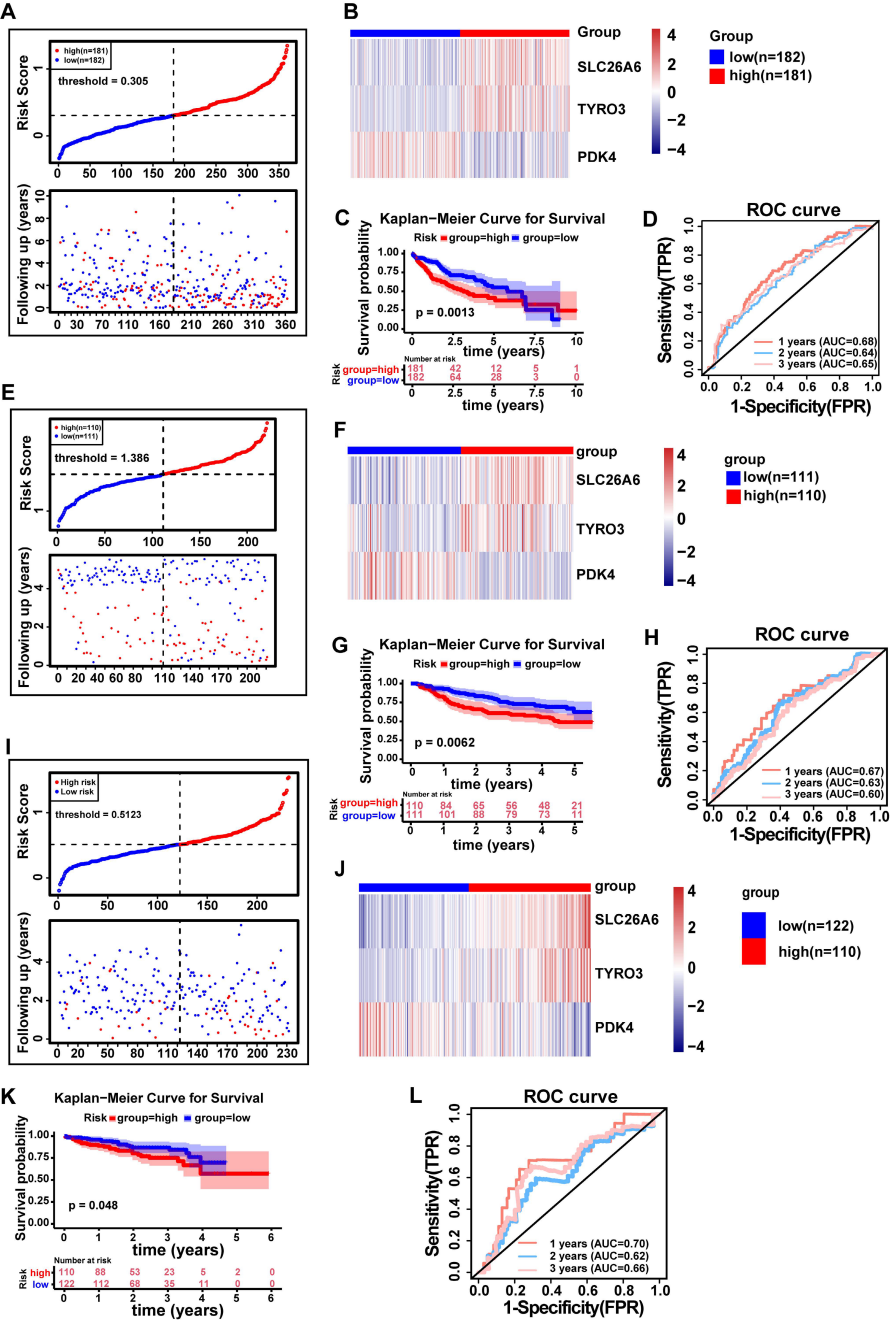
The risk model was established to assess the prognostic risk of HCC patients. **A.** Risk curve (TCGA-LIHC). **B.** Heat map of HCC prognostic gene expression in high and low risk groups (TCGA-LIHC. C. E. KM curve of high and low gene expression (SLC26A6). **C.** KM survival curve (TCGA-LIHC). **D.** Time-dependent ROC curve (TCGA-LIHC). **E.** Risk curve (GSE14520 validation set). **F.** Heatmap of prognostic gene expression for HCC in high and low risk groups (GSE14520 validation set). **G.** KM survival curve (GSE14520 validation set). **H.** Time-dependent ROC curve (GSE14520 Validation Set). **I.** Risk curves (ICGC-HCC validation set). **J.** Heatmap of HCC prognostic gene expression in high and low risk groups (ICGC-HCC validation set).** K.** KM Survival Curve (ICGC-HCC Validation Set). **L.** Time-dependent ROC curve (ICGC-HCC Validation Set). ROC, receiver operating characteristic curve. FPR, False positive rate. TPR, True positive rate. KM Kaplan-Meier.

We constructed a risk model using prognosis genes, with Risk Score calculated as follows: Risk Score=SLC26A6*0.2575+TYRO3*0.1602+PDK4*(-0.0781). The specific calculation method for the risk score is described in **Materials and methods 2.5**. HCC patients were stratified into distinct risk groups via median value of the risk score. The risk scores were sorted from low to high, and a risk plot was generated (**Fig. 4A**). The patients with high risk exhibited more deaths than patients with low risk, with samples in the former displaying shorter survival times than the latter. Hence, it could be inferred that patients with low risk scores in TCGA-LIHC dataset had a better survival prognosis. And we also demonstrated the expression of prognosis genes in distinct risk groups (**Fig. 4B**). As anticipated, patients classified as low risk demonstrated significantly longer survival compared to those classified as high risk (*P* < 0.01) (**Fig. 4C**). The model was evaluated using time-dependent ROC analysis, with AUC of 0.68, 0.64 and 0.65 at 1, 2 and 3 years, respectively, suggesting that our risk model demonstrated a strong predictive ability for assessing the risk of HCC patients (**Fig. 4D**). Moreover, we conducted verifications of our risk model's predictive value in both the GSE14520 and LIRI-JP datasets. Importantly, the results obtained were consistent with those observed in TCGA-LIHC dataset (AUC > 0.6 and *P* < 0.05) (**Fig. 4E-L**). The findings suggested that our risk model was highly proficient in assessing the risk of HCC patients.

### 3.5 Risk score and pathologic T stage were independent factors of HCC prognosis

**Figure 5:**
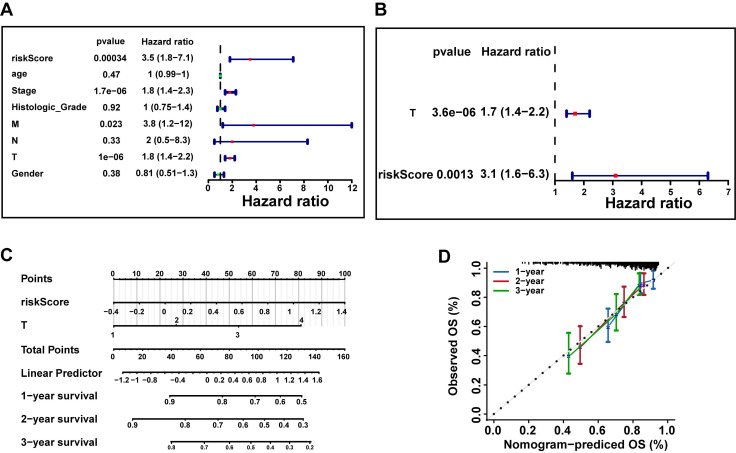
Construction of independent prognostic models based on the TCGA-LIHC Dataset. **A.** Univariate Cox regression analysis of risk scores and clinical characteristics (age, gender, stage, histological grade, and pathological TNM stage) in the TCGA-LIHC dataset (P < 0.05 and HR ≠ 1). **B.** Multivariate Cox regression analysis identified independent prognostic factors (P < 0.05 and HR ≠ 1) based on univariate Cox regression results. **C.** Nomogram predicting 1-, 2-, and 3-year survival rates for HCC patients based on risk score and pathologic T stage. **D.** The calibration curves were used to assess the predictive performance of the nomogram. The dashed line shows the ideal relationship between observed and predicted OS rates. If all data points fall on this line, the prediction is very accurate.

To further investigate whether risk scores could function as an independent factor for predicting the prognosis of patients with HCC. Hence, we incorporated risk scores and clinical characteristics into univariate and multivariate Cox regression analysis in TCGA-LIHC dataset. Firstly, univariate Cox regression analysis of risk scores and clinical characteristics in the TCGA-LIHC dataset confirmed that risk score, stage, M stage, and T stage are significantly associated with the prognosis of HCC patients (P < 0.05 and HR≠1) (**Fig. 5A**). Subsequently, based on the results of the multivariate Cox regression analysis, we identified only two independent prognostic factors: risk score and pathological T stage (P < 0.05) (**Fig. 5B**). Eventually, we constructed a nomogram to predict survival rate of HCC patients at 1, 2, and 3 years via risk score and pathologic T stage (**Fig. 5C**). The calibration curves demonstrated that the nomogram exhibited favorable predictive accuracy, indicating its great potential for clinical application (**Fig. 5D**).

### 3.6 Significant differences in risk scores, clinical characteristics, and survival status among subgroups of HCC patients

**Figure 6:**
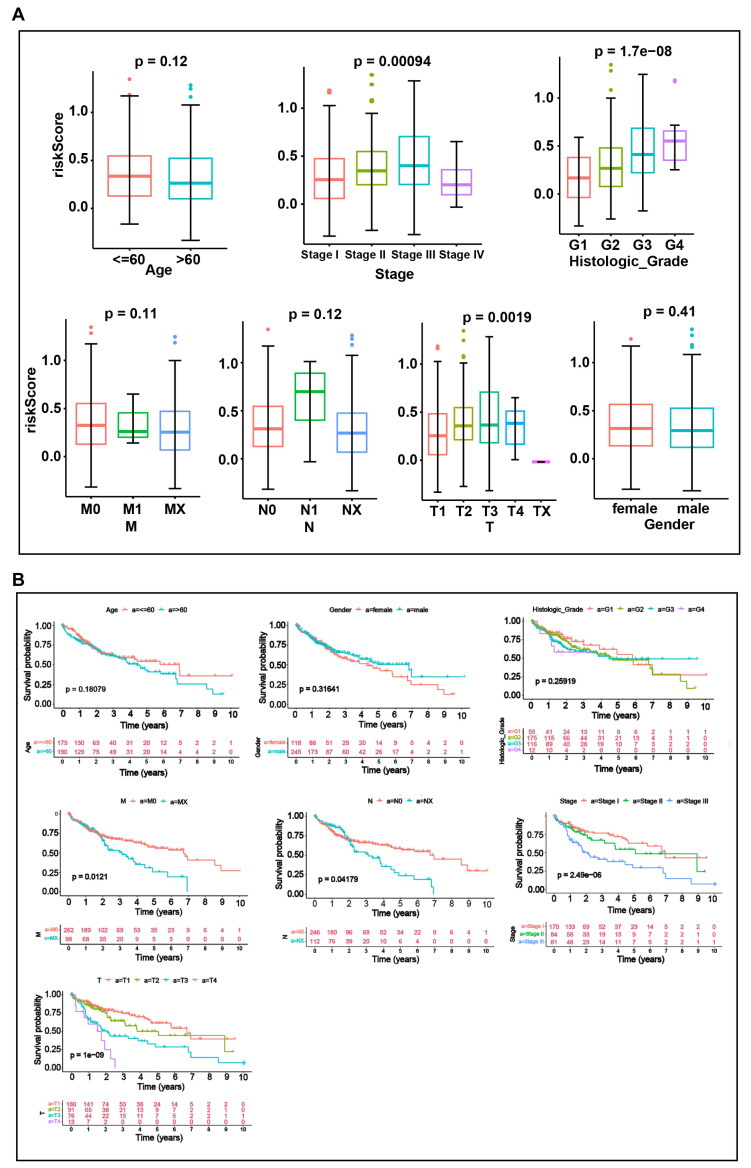
Analysis of the relationship between risk scores and clinical characteristics. **A.** Boxplots of risk scores across clinical characteristics (Age, Stage, histologic grade, Gender, T, M, N). **B.** KM curves between subgroups of different clinical characteristics (Age, Stage, histologic grade, Gender, T, M, N). Differences between groups were analyzed by Wilcoxon test. P < 0.05 was considered statistically significant.

To explore the relationship between risk scores and clinical characteristics, we performed an analysis across different clinical subgroups. Risk scores displayed significant differences among subgroups of the three clinical indicators: stage, histologic grade, and pathologic T stage (*P* < 0.05) (**Fig. 6A**). And significant differences in survival status were observed among subgroups for each of the four clinical characteristics: Stage (stage1 and stage 2, stage 3), T (T1 and T2, T3), N (N0 and Nx) and M (M0 and Mx) subgroups (**Fig. 6B**). The risk score is calculated based on the expression of multiple prognosis-related genes. Although the risk score shows significant differences across different clinical subgroups, its predictive value for survival status may vary due to the interactions among these factors.

### 3.7 The low risk group demonstrated enrichment in pathways related to biometabolism and immune response

**Figure 7:**
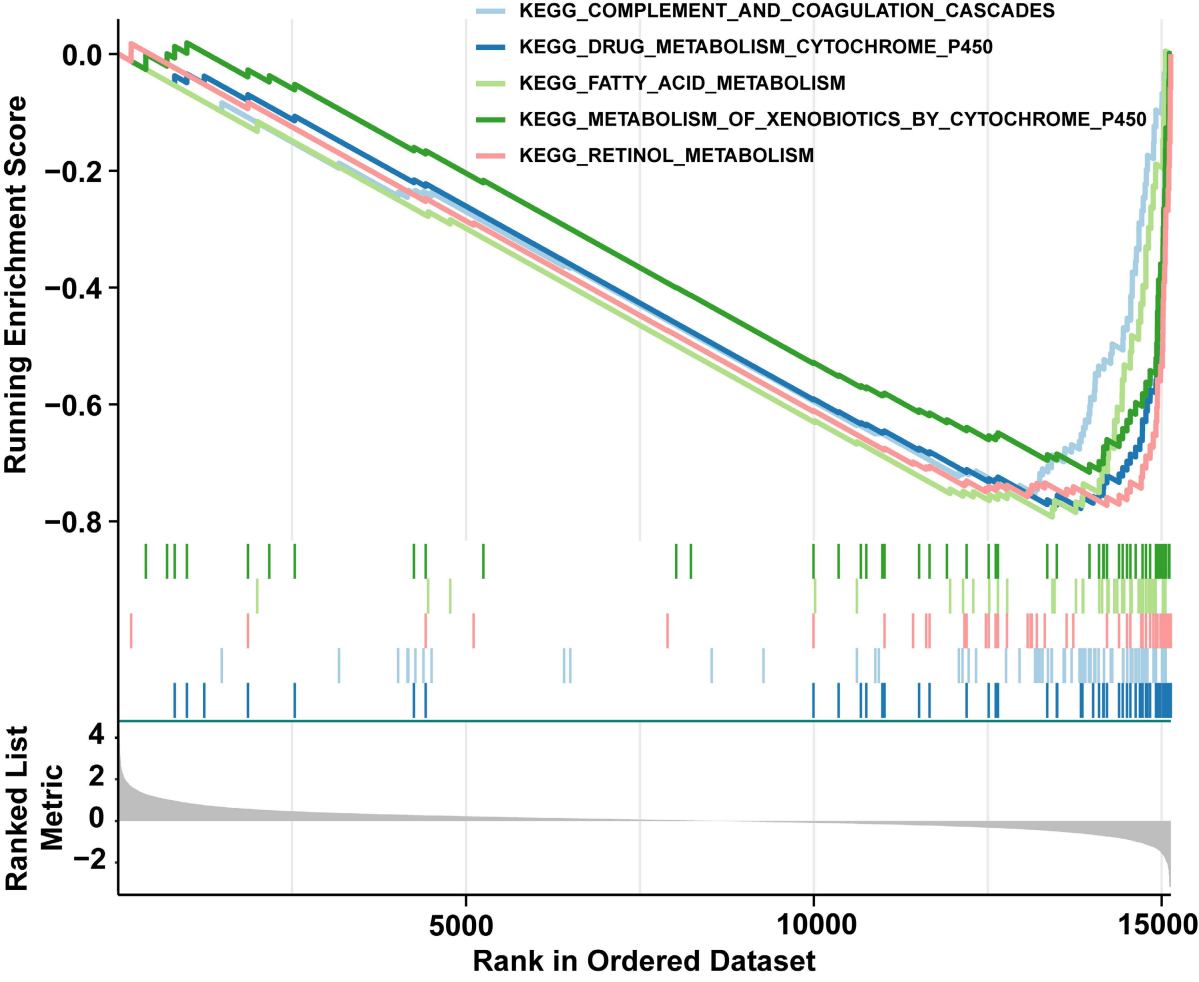
GSEA enrichment analysis of differentially expressed genes among distinct risk groups. This figure shows the GSEA enrichment results of differentially expressed genes in the top five KEGG pathways, mainly concentrated in the low-risk group (right side of the curve peaks).

Next, GSEA was constructed to explore relevant signaling pathways and potential biological mechanisms associated with the presence of distinct risk groups. To be specific, we observed that low risk group was chiefly enriched in "complement and coagulation cascades", "drug metabolism cytochrome p450", "fatty acid metabolism", "metabolism of xenobiotics by cytochrome p450", and "retinol metabolism"(**Fig. 7 and Table S3**). Hallmark conducted verification and enrichment of 18 pathways, among which two pathways were the same, namely "fatty acid metabolism". The others were highly similar to KEGG (**Fig. S1 and Table S4**). The findings demonstrated that group at low risk exhibited enrichment in pathways associated with biometabolism and immune response, which were pivotal in governing the body's response to injury and infection, drug metabolism and detoxification, maintenance of energy homeostasis, cellular structure and signaling, as well as vitamin A metabolism and function. These interconnected signaling pathways collectively participate in numerous vital physiological processes, thus augmenting our comprehension and knowledge of HCC.

### 3.8 Prognostic genes reflect the immune status in the HCC tumor microenvironment (TME), and the risk score predicts immunotherapy response

**Figure 8:**
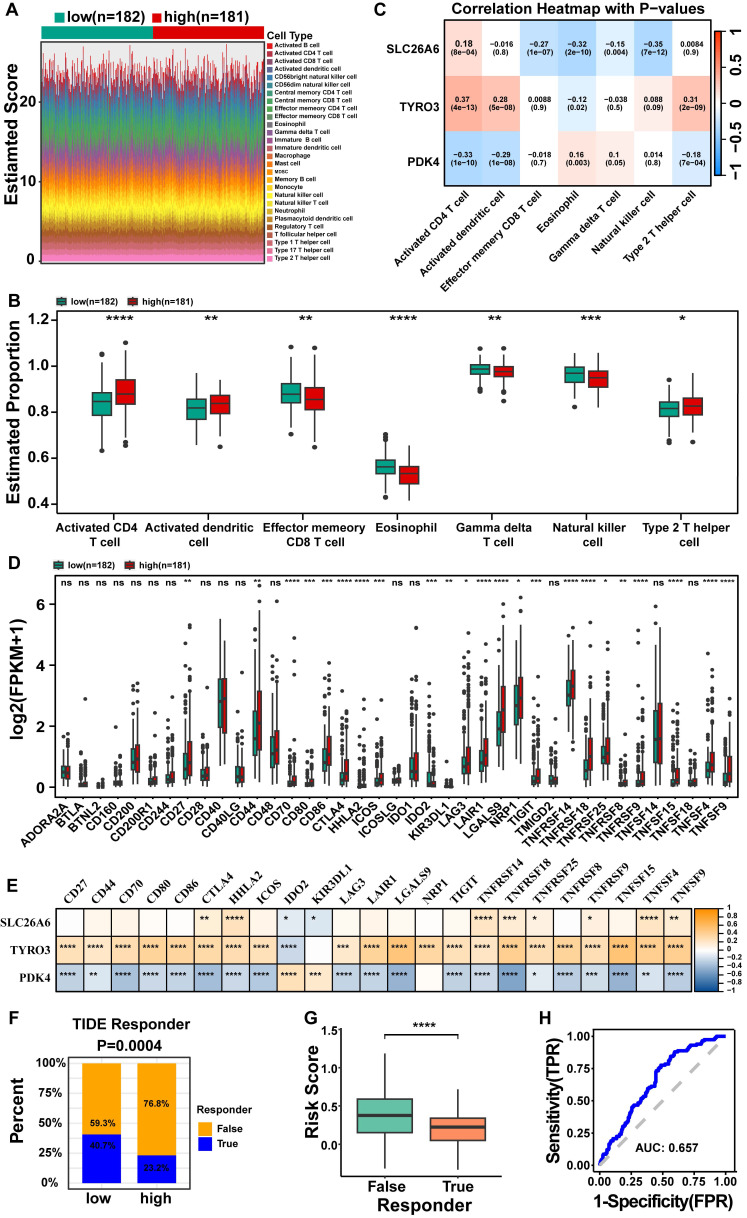
Analysis of differences in immune cell infiltration and immunotherapy response among different risk groups of HCC patients in the TCGA-LIHC dataset.** A.** Heat map of 28 mmune cell infiltration. **B.** The box line plot demonstrates that 7 out of 28 immune cell infiltrates exhibited statistically significant differential expression between high- and low-risk groups, with the y-axis representing expression levels of signature genes. **C.** Heat map of HCC prognostic genes correlating with differential immune cells.** D.** Box plots of immune checkpoint gene expression in high and low risk groups. **E.** Heat map of HCC prognostic genes correlating with 23 immune checkpoint molecules. **F.** Immunotherapy response or non-response ratio between high and low risk groups.** G.** Differences in risk scores between the groups that responded or did not respond to immunotherapy. **H.** ROC curves of risk scores versus response to immunotherapy. The statistical analysis employed the Wilcoxon test. Data were presented as mean ± SD. ns, not significant; *, p<0.05; **, p<0.01; ***, p<0.001; ****, p<0.0001.

The results obtained from GSEA indicated a significant association between distinct risk groups and immune response-related signaling pathways. These findings prompted us to further investigate the role of the immune microenvironment in HCC. We visually represented the abundance of 28 immune cells infiltration in distinct risk groups through heatmap (**Fig. 8A**). Noteworthy, activated CD4^+^ T cell, activated dendritic cell, and Type 2 T helper cell (Th2 cell) were significantly higher in group at high risk (*P*<0.05), the opposite was true for effector memory CD8^+^ T cell, eosinophil, gamma delta T cell (γδ T cells), and natural killer cell (NK) (*P*< 0.05) (**Fig. 8B**). The results of spearman correlation analysis revealed a strong positive correlation between activated CD4^+^ T cells and TYRO3 (cor = 0.37, P < 0.0001) and a negative correlation with PDK4 (cor = -0.33, P < 0.0001). Additionally, NK cells exhibited the strongest negative correlation with SLC26A6 (cor = -0.35, P < 0.0001)** (Fig. 8C)**. The findings suggested that prognosis genes might exert influence on the immune response among patients with HCC, possibly through variations in their expression levels. Consequently, our prognostic model not only held promise as an effective tool for assessing the immune response status, but also offered valuable insights and directions for the advancement of novel immunotherapeutic approaches.

Besides, we evaluated the expression levels of 39 immune checkpoints from distinct risk groups in TCGA-LIHC dataset (**Fig. 8D**). A total of 23 immune checkpoint molecules exhibited significant differences from distinct risk groups, encompassing CD27, CD44, CD70, CD80, CD86, CTLA4, HHLA2, ICOS, IDO2, KIR3DL1, LAG3, LAIR1, LGALS9, NRP1, TIGIT, TNFRSF14, TNFRSF18, TNFRSF25, TNFRSF8, TNFRSF9, TNFSF15, TNFSF4, and TNFSF9 (*P* < 0.05) (**Fig. 8D**). The analysis of prognostic genes and differential immune checkpoints revealed that TNFSF15 was strongly positively correlated with TYRO3 (cor = 0.53, p = 1.61E-27), but negatively correlated with PDK4 (cor = -0.42, p = 1.29E-16) (**Fig. 8E and Table S5**). The response of HCC patients to immunotherapy was evaluated through TIDE database. The results indicate that HCC patients in the high risk group exhibit significantly lower responsiveness to immunotherapy compared to those in the low risk group. Specifically, only 23.2% of high risk patients responded, while 40.7% of low risk patients responded, with the difference being statistically significant (P = 0.0004) (**Fig. 8F**). There is a significant difference in risk scores between the TRUE (Responder) and FALSE (Non-responder) groups (P < 0.05), with non-responder exhibiting a higher median risk score compared to responder (**Fig. 8G**). This suggests that a higher risk score may be associated with poorer treatment responsiveness. The ROC curve analysis yielded an AUC of 0.657 (**Fig. 8H**), further underscored the strong predictive capacity of risk scores in determining the response to immunotherapy.

### 3.9 Mutations in prognosis genes were found to have a significant impact on HCC and displayed a correlation with immune cell infiltration

**Figure 9:**
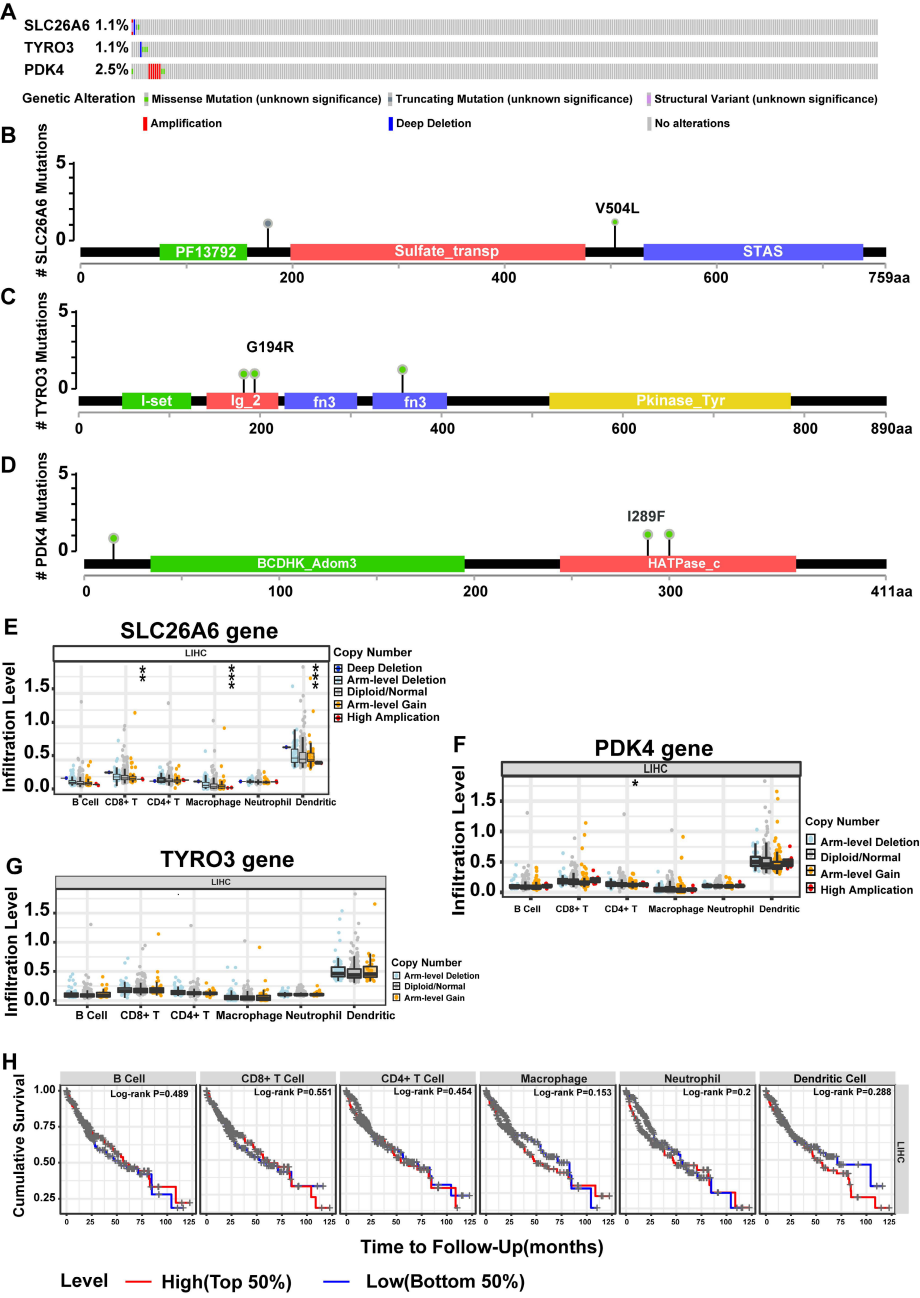
Mutations in prognosis-related genes significantly impacted HCC and correlated with immune cell infiltration in the TCGA-LIHC dataset. **A.** Overall view of genetic variation in 3 HCC prognostic genes. **B.** Map of SLC26A6 structural domain mutations. **C.** Map of TYRO3 structural domain mutations. **D.** Mapping of PDK4 structural domain mutations.** E.** HCC prognostic gene for immune cell infiltration (SLC26A6). **F.** HCC prognostic gene immune cell infiltration (TYRO3). **G.** HCC prognostic gene immune cell infiltration (PDK4). H. KM curves of high and low immune cell infiltration levels. The statistical analysis employed the Wilcoxon test. Data were presented as mean ± SD. *p < .05, **p < .01, ***p < .001.

The SLC26A6 showed mutations in 1.1% of HCC patients, while the TYRO3 exhibited mutations in 1.1%, and the PDK4 had mutations in 2.5% (**Fig. 9A**). Notably, the predominant mutation type observed in the PDK4 was copy number increase (amplification). Furthermore, the structural maps of functional domains for prognosis genes depict the specific mutation sites (**Fig. 9B-D**). Concurrently, we examined the correlation between copy number variation (CNV) of prognosis genes and the extent of immune cell infiltration. Significant differences were observed in the levels of CD8^+^ T cells, macrophages, and DCs across different CNV states of SLC26A6 (P < 0.01) (**Fig. 9E**), while CD4^+^ T cell levels showed significant variation among the CNV states of PDK4 (**Fig. 9F**) (P < 0.05). In contrast, immune cell infiltration levels did not differ significantly across the CNV states of TYRO3 (**Fig. 9G**). Next, based on the median infiltration levels of immune cells in the TME, the patients were divided into high-infiltration and low-infiltration groups. However, we did not observe a significant impact of the infiltration levels of these immune cells on the OS of HCC patients (*P* > 0.05) (**Fig. 9H**). This section highlights the complex interactions between DE-ERGs gene mutations, immune cell infiltration in the TME, and HCC prognosis. While CNVs of DE-ERGs genes affect immune cell infiltration, their impact on OS may involve complex immune regulatory mechanisms. Given the study's sample size limitations, further mechanistic research and multicenter clinical follow-up are needed to clarify these relationships.

### 3.10 Construction of lncRNA-miRNA-mRNA network provided insights into the potential regulatory mechanisms underlying prognosis-related gene expression in HCC

**Figure 10:**
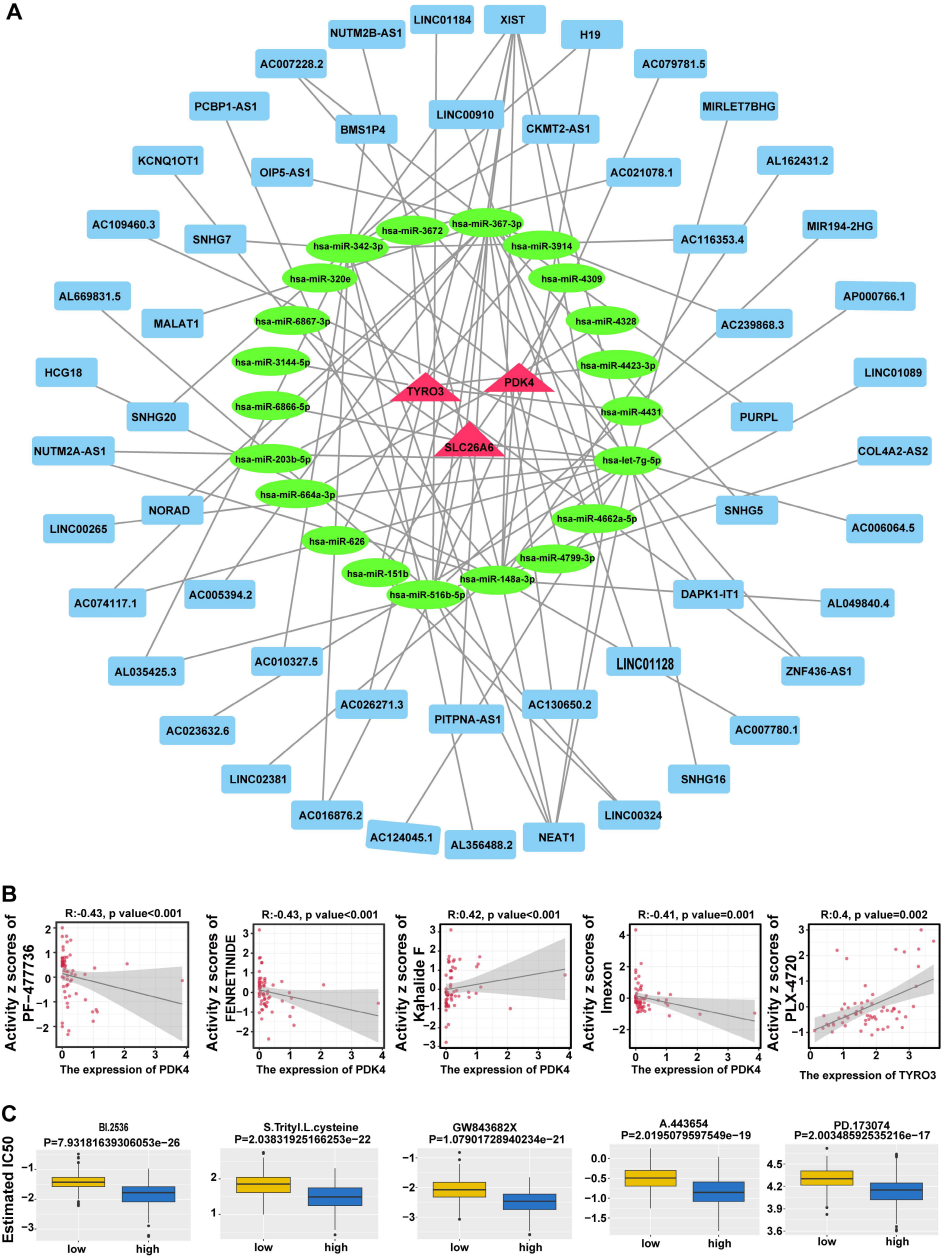
Construction of competing endogenous RNA (ceRNA) network and drug sensitivity analysis. **A.** Prognostic gene ceRNA network with red triangles for mRNAs, green circles for miRNAs, and blue rectangles for lncRNAs. **B.** Prognostic gene expression correlation with drug sensitivity. **C.** Difference in drug IC50 between high and low risk groups.

A total of 21 miRNAs corresponding to prognosis genes were predicted in miRWalk database, while starBase database predicted 7 miRNAs corresponding to 53 lncRNAs. Based on these findings, we constructed a lncRNA-miRNA-mRNA network demonstrating 53 lncRNA regulated three mRNAs through 21 miRNAs (**Fig. 10A**). Totally 92 interaction pairs were formed, such as NUTM2A-AS1-hsa-miR-203b-5p-TYRO3, AC006064.5-hsa-let-7g-5p-SLC26A6 and XIST-hsa-miR-367-3p-PDK4. The results indicated the regulatory mechanism for prognosis genes in HCC.

### 3.11 Analysis of drug sensitivity revealed potential therapeutic avenues for HCC

In addition, 860 drugs were identified through screening, showing the top 5 correlations between the expression of prognosis genes and drug sensitivity data** (Fig. 10B)**, including PF-47736, PENRETINIDE, Kahalide F, Imexon, and Plx-4720. Most of these drugs are currently in preclinical research or early clinical trial stages and show promise for the treatment of cancer and other diseases. In total, 61 drugs showed significant differences in IC_50_ values between distinct risk groups (*P* < 0.05) (**Fig. S2**). The top 5 drugs, comprising BI.2536, S-Trityl-L-cysteine, GW843682X, A-443654, and PD-173074, were presented in descending order of *P*-value (**Fig. 10C**). These drugs played a crucial role in the realm of cancer research and treatment, offering invaluable insights for the advancement of drug discovery and development. And these findings highlighted potential therapeutic avenues and further emphasized the significance of prognosis-related gene expression in HCC.

### 3.12 Hepatocyte were regarded as key cells

**Figure 11:**
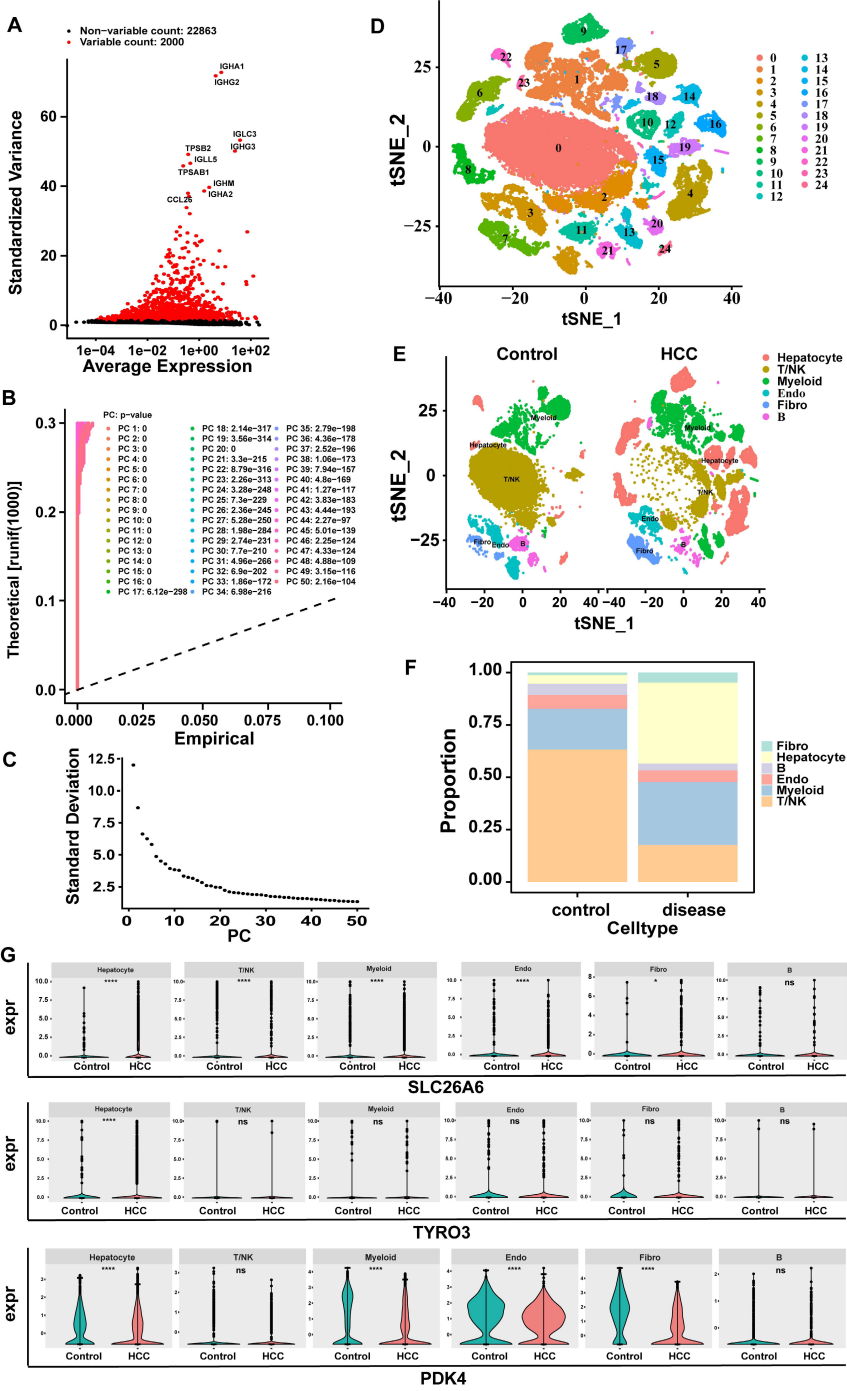
Screening of key cells. **A**. Screening for highly variable genes (hvg): red indicates highly variable genes, and black represents non-highly variable genes. **B-C**:30 PCs were attained in this study for subsequent analysis. **D**. Cell tSNE clustering map (Each cluster number from 0 to 24 was assigned to 25 independent cell populations, with each number representing a unique cell cluster). **E**. tSNE clustering map of cells grouped by control diseases. **F**. Cell proportion bar stack chart. **G**. Violin plots of key gene expression levels (SLC26A6, TYRO3, PDK4).

In the GSE149614 dataset, after QC, 24,863 genes and 57,459 cells met the criteria (**Fig. S3A-B**). Subsequently, after data normalization processing, we discovered 2000 highly variable genes (hvg) and marked the top 10 hvg (**Fig. 11A**). After further narrowing down the data, 35 principal components (PCs) were obtained in this study for subsequent analysis (p < 0.05) (**Fig. 11B-C**). Subsequently, based on cell cluster analysis, 25 different cell clusters were revealed, including Cluster 0 to Cluster 24 (**Fig. 11D**). Next, based on the marker genes of SingleR, these cell clusters were further annotated into six cell types: T/NK (T cells and natural killer cells), hepatocytes (hepatocytes), myeloids (Myeloid cells), B (B cells), endo (endothelial cells), Fibro (fibroblasts) (**Fig. 11E**). The cell proportion bar stack graph shows the high specificity of the marker gene, confirming the accuracy of the annotation (**Fig. 11F**). Subsequently, the Wilcoxon test was used to compare the expression differences of key genes in the annotated cell types. The key gene SLC26A6 showed significant expression differences in T/NK (T cells and natural killer cells), hepatocytes (hepatocytes), myeloids (Myeloid cells), endo (endothelial cells), and fibro (fibroblasts) respectively. The key gene TYRO3 had significant expression differences in hepatocytes. The key gene PDK4 had significant expression differences in hepatocytes, myeloids, Endo (endothelial cells), and Fibro (fibroblasts). The cells with significant expression differences in all three key cells are hepatocytes (**Fig. 11G**). Therefore, hepatocytes were selected as the key cells.

### 3.13 Pseudo-time trajectory analyses

**Figure 12:**
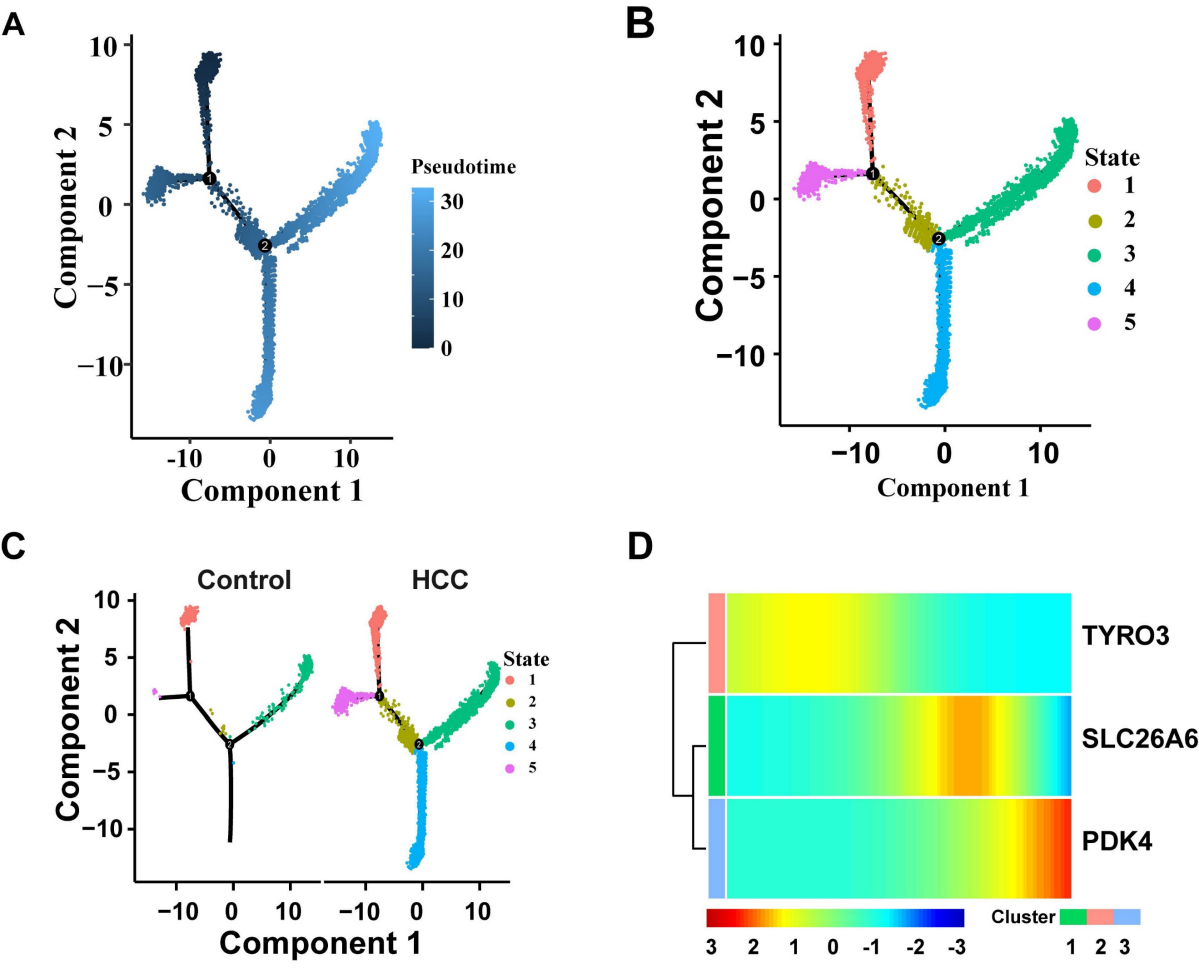
Pseudo-time trajectory analyses. **A**. Cell differentiation trajectory map. **B**. Diagram of cell differentiation status. **C**. Cell differentiation status maps of HCC and the control group. **D.** The heat map of dynamic expression of prognostic genes in hepatocytes. The color bands in the figure represent the levels of gene expression. The redder the color, the higher the expression level, and the bluer the color, the lower the expression level.

Hepatocytes go through three key nodes during their development and differentiation. State1 is at the starting point of differentiation of tepatocytes, state2 is in the process of hepatocyte differentiation and is a key transitional state connecting the initial differentiation and the middle differentiation; State3 and State5 are in the middle stage of differentiation of tepatocytes, and State4 and State3 are in the end stage of differentiation of tepatocytes (**Fig. 12A-B**). Compared with the control group, the HCC group exhibited a significant skewing in the proportions of specific differentiation states (e.g., State 4 and State 3) (**Fig. 12C**). This perturbation in the differentiation landscape suggests that hepatocarcinogenesis is associated with a dysregulated hepatocyte differentiation program. The expression changes of three prognostic genes over time were detected. The results showed that the expression level of TYRO3 in hepatocytes decreased slowly along the time trajectory, and the expression level of SLC26A6 in hepatocytes along the time trajectory increased in the middle of differentiation and then gradually decreased over time. The expression level of PDK4 gradually increased in hepatocytes over time (**Fig. 12D**). These findings suggest that TYRO3 function appears to be predominant in the early differentiation stage, SLC26A6 may participate in metabolic and signal transduction processes during the mid-differentiation phase, while PDK4 could potentially regulate energy metabolism in the terminal differentiation stage. Pseudotime trajectory analysis not only reveals the dynamic expression patterns of SLC26A6, TYRO3, and PDK4 during hepatocyte differentiation but also provides a temporal context for understanding the roles of these genes in hepatocellular carcinoma development.

### 3.14 Analysis of cell communication revealed intercellular ligand-receptor interactions

**Figure 13:**
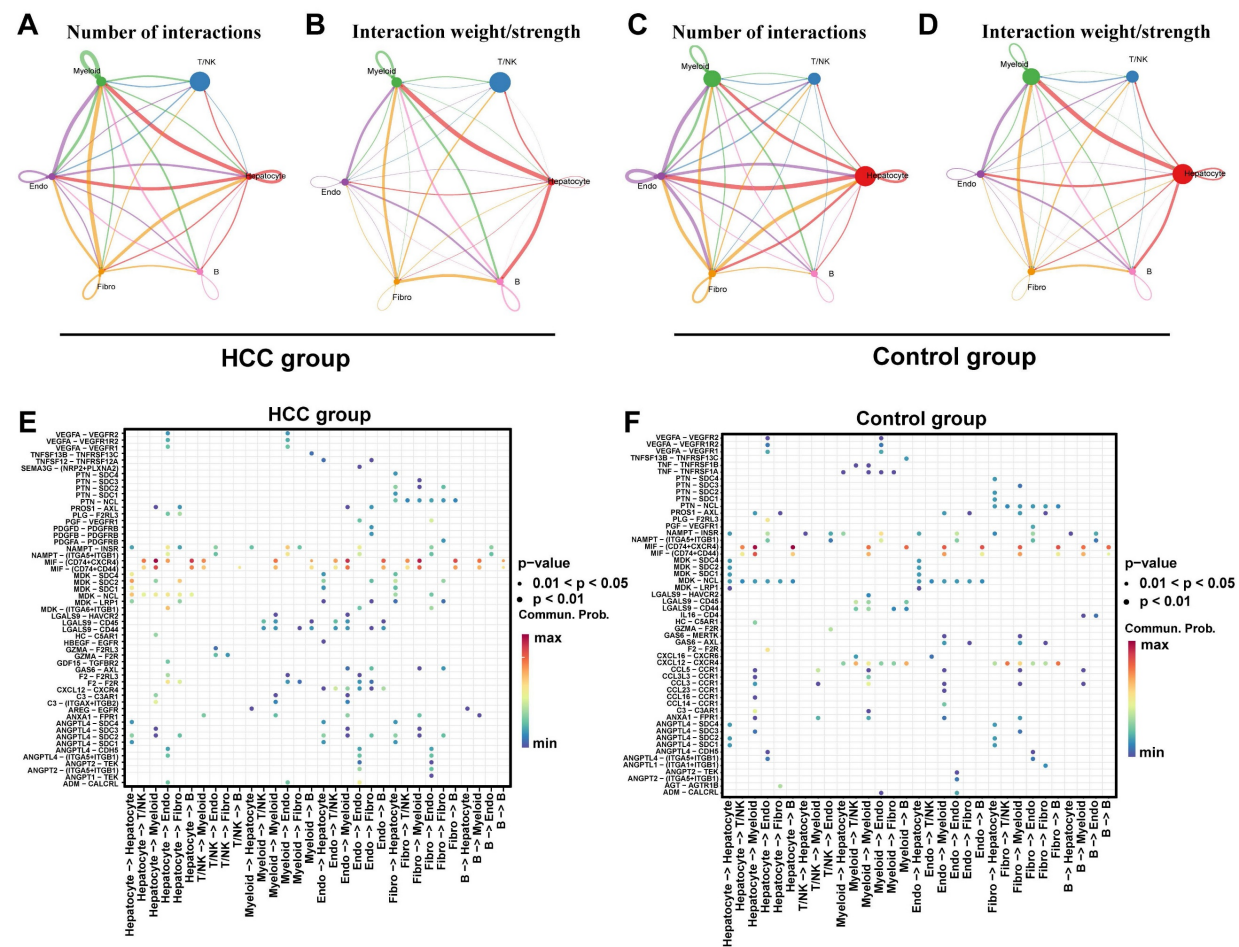
Analysis of cell communication.** A.** The cell communication frequency in the HCC group, the connection lines represent the communication relationship between cells, and different colors distinguish the types of cell communication. **B.** Cell communication intensity map of the HCC group. **C.** The cell communication frequency in the control group. **D.** Cell communication intensity map of the control group.** E**. The receptor-ligand pair map of intercellular interactions in the HCC group. **F**. The receptor-ligand pair map of intercellular interactions in the control group.

Cell - cell communication analysis focuses on studying the interactions between key cells and other cells. Specifically, the results of the intercellular communication network showed that in the HCC group and control group, the cell communication between Hepatocyte and Myeloid cells, Endo (endothelial cells), and Fibro (fibroblasts) was more frequent, and the cell communication intensity between Hepatocyte and Myeloid, as well as between Hepatocyte and B cells was relatively high (**Fig. 13A-D**). The signaling molecules for intercellular communication among various cells in the HCC group showed that the receptor - ligand interactions with relatively strong effects between hepatocytes and both myeloid and B cells were MIF-(CD74+CXCR4) and MIF-(CD74+CD44) (**Fig. 13E**). In the control group, the receptor - ligand interaction with a relatively strong effect between hepatocytes and B cells was MIF-(CD74+CXCR4). The receptor - ligand interaction with a relatively strong effect between hepatocytes and myeloid cells were MIF-(CD74+CXCR4) and MIF-(CD74+CD44) (**Fig. 13F**). These findings demonstrate a significant enhancement of MIF-(CD74+CXCR4) and MIF-(CD74+CD44) receptor-ligand interactions in HCC compared to controls. The MIF pathway, known to activate downstream signaling through CD74 binding, plays crucial roles in inflammation, cell migration, and immune regulation during disease and cancer progression^42^, ^43^. Prior evidence linking TYRO3 and PDK4 to the immune checkpoint CD44 (**Fig. 8E**) suggests that MIF-(CD74+CXCR4) and MIF-(CD74+CD44) may cooperate with SLC26A6, TYRO3, and PDK4 in modulating HCC progression and immune microenvironment remodeling.

## 4. Discussion

HCC is characterized by rapid progression, substantial clinical treatment expenses, and a significant healthcare burden, resulting in less than optimal therapeutic outcomes. Despite ongoing research and therapeutic innovations, the prognosis for patients with advanced-stage HCC continues to be discouragingly poor^44^. Since its initial characterization by deCathelineau AM et al. in 2003, efferocytosis has emerged as a pivotal area of focus in oncological research, attributed to its essential roles in modulating cancer cell survival, orchestrating the tumor microenvironment (TME), and influencing tumor progression^45^. This study aims to explore the potential clinical value of ERGs in the prognosis and treatment of HCC. The goal is to provide a novel theoretical basis for predicting tumor progression and facilitating the development of new therapeutic strategies. In summary, this study identified three efferocytosis-related prognostic genes (SLC26A6, TYRO3, and PDK4) and integrated them into a highly effective prognostic risk model, offering novel insights into the prognosis of HCC. Furthermore, we found that these prognostic genes have significant value in predicting the response to immunotherapy and guiding clinical treatment for HCC. This can aid clinicians in developing more personalized treatment plans based on gene expression profiles, ultimately improving therapeutic outcomes and enhancing patient quality of life.

Recent research indicates that the TYRO3 gene is significantly upregulated (more than twofold) in the tumor tissues of approximately 42% of HCC patients. This overexpression of TYRO3 not only suppresses the transcription and translation of the HCC tumor marker alpha-fetoprotein (AFP) but also, when silenced, reduces cell proliferation, ERK phosphorylation, and cyclin D1 expression, suggesting TYRO3's role as a tumor suppressor in HCC^46^. Moreover, clinical studies have linked TYRO3 with increased hepatitis activity and poor prognosis, proposing that its expression promotes HCC progression in the context of active hepatitis. Experimental models further validate this by showing that inhibiting TYRO3 expression or kinase activity curtails the growth of HCC xenografts in nude mice^47^. Additionally, Song F et al. have identified a correlation between PDK4 expression in HCC and abnormal tumor cell metabolism, although more research is needed to elucidate the underlying mechanisms^48^. The absence of PDK4 expression, as shown by Qin YJ et al., accelerates the malignant progression of HCC^49^. Concurrently, other studies have highlighted that miR-9-5p enhances mitochondrial energy metabolism in HCC cells by downregulating PDK4, which contributes to the progression of the disease. Both miR-9-5p and PDK4 are considered potential therapeutic targets for preventing HCC recurrence and metastasis^50^. The suppression of PDK4 has also been shown to increase the expression of key lipogenic enzymes such as fatty acid synthase (FASN) and stearoyl-CoA desaturase (SCD), ultimately inducing lipogenesis, which suggests that PDK4 may inhibit the proliferation and migration of HCC cells by suppressing lipogenesis^51^. In further research, Zhang et al. revealed that G9a and Suv39H facilitate HCC progression via H3K9 methylation-mediated silencing of PDK4, with arsenic exposure exacerbating this effect^52^. A pivotal study have confirmed that SLC26A6 is upregulated in HCC and associated with poor prognosis, demonstrating overexpression at both mRNA and protein level^53^. The knockout of SLC26A6 suppresses HCC progression, suggesting its potential as a therapeutic target, with bioinformatics analysis revealing its involvement in multiple cancer-related pathways^54^. Additionally, SLC26A6 has been recognized as a critical gene for drug metabolism in NAFLD^55^. The distinct roles of the three prognostic genes in HCC reflect the complexity of the disease. Their expression and functional changes may significantly impact the onset, progression, and TME of HCC. A deeper understanding of the mechanisms underlying these genes' actions will contribute to the development of new therapeutic strategies.

Tumor growth is significantly influenced by efferocytosis, which plays a crucial role in physiological balance and disease pathogenesis. In HCC tissues, tumor cells continuously proliferate, undergo apoptosis, and die, releasing various cytokines and chemical substances. This process triggers inflammatory responses and immune reactions, leading to the recruitment of macrophages, DCs, and NK cells. These immune cells regulate inflammation and immune responses by clearing apoptotic cells^56^-^58^. However, emerging research highlights the dual role of efferocytosis in tumor progression, revealing its complex influence on cancer biology. On one hand, tumor cells strategically exploit efferocytosis to evade immune surveillance by expressing specific receptors and ligands associated with this process^7^. These molecules attract immune cells to clear apoptotic cells in the TME, inadvertently shielding the tumor cells from immune attacks^7^, ^59^-^61^. Conversely, modulating the efferocytosis process can amplify immune cell-mediated tumor clearance and promote tumor cell apoptosis, thereby significantly impeding tumor growth and metastasis^62^. Therefore, a deeper understanding of the intricate interplay between efferocytosis and tumor dynamics is crucial for advancing the development of more effective cancer therapies.

Our risk model has significant clinical value in prognostic prediction. Compared with the low-risk group, patients in the high-risk group have a poorer prognosis. With an AUC > 0.6, the model demonstrates good predictive performance. Both univariate and multivariate Cox regression analyses confirmed that the risk score is an independent predictive factor for HCC patients. Additionally, a nomogram was constructed using the risk score and T stage. The calibration curve indicates that the nomogram has high predictive accuracy. This provides a new model for the prognostic prediction of HCC, but the clinical applicability of the model still needs to be validated.

In this study, we constructed a risk prediction model for HCC based on SLC26A6, TYRO3, and PDK4, aiming to evaluate the roles of these ERGs in HCC prognosis. GSEA revealed that patients in the low-risk group exhibited significant enrichment in pathways including the complement and coagulation cascades, fatty acid metabolism, and vitamin A metabolism. Notably, the complement and coagulation cascades pathway is directly linked to efferocytosis, as complement components (e.g., C1q, C3, and C4) opsonize apoptotic cells to facilitate their recognition and phagocytosis by macrophages and other phagocytes—a core mechanism of efferocytosis. Dysregulated activation or suppression of the complement system may impair apoptotic cell clearance efficiency, thereby influencing inflammatory responses and tissue repair processes. These findings align with the results of our study^63^-^65^. Furthermore, the "fatty acid metabolism" and "vitamin A metabolism" pathways indirectly influence efferocytosis by modulating cellular energy metabolism and signal transduction^66^-^68^. Fatty acid metabolites, such as prostaglandins and leukotrienes, can modulate immune cell activity and inflammatory responses. Meanwhile, retinoic acid signaling plays a crucial role in regulating immune cell differentiation and function, thereby indirectly affecting the phagocytic capacity of phagocytes^69^, ^70^. Collectively, these results reveal the critical role of efferocytosis in HCC pathogenesis, support the rationale for ERG-based risk stratification, and offer important insights into efferocytosis function during disease progression. Consequently, our findings lay the groundwork for subsequent mechanistic studies and clinical applications. Taken together, the risk model established here partially reflects the mechanistic involvement of efferocytosis in HCC, providing a foundation for further in-depth investigations.

In the current study, the low-risk group of HCC patients was predominantly enriched in pathways related to metabolism and immune responses. As an essential component of the innate immune system, the complement system is present in the TME and plays a dual role in tumor biology. It has been reported that elevated expression levels of complement component 8 beta (C8B) have a protective effect on OS and recurrence-free survival (RFS) in patients with HBV-associated HCC^65^. Another study suggested that HCC with higher levels of inflammatory responses is enriched in immune-related signaling pathways, including the complement and coagulation cascades, indicating that inflammation-prone HCC exhibits an enhanced immune response^71^. Human cytochrome P450 (CYP) enzymes play a critical role in drug detoxification^72^, cellular metabolism and maintaining homeostasis^73^. Approximately 80% of oxidative metabolism and 50% of the clearance of commonly used clinical drugs are attributed to one or more enzymes from the CYP 1-3 family^72^. In cancer, the expression of drug-metabolizing CYPs can be aberrant, such as the overexpression of CYP1B1 in breast cancer cells^74^ and CYP2A6 in liver^75^ and lung cancers^76^. Due to these expression abnormalities, CYPs have been identified as potential targets and biomarkers for anti-cancer therapies. In this study, the low-risk HCC group showed negative enrichment for drug metabolism cytochrome P450, suggesting the need for further investigation into how SLC26A6, TYRO3, and PDK4 affect CYP enzymes and their role in drug sensitivity during HCC treatment. These genes may serve as new therapeutic targets for HCC. Fatty acid metabolism plays a crucial role in HCC, regulating cancer cell metabolism and energy supply, and promoting proliferation and survival^77^. It also affects the tumor's immune microenvironment. For instance, tumor-associated macrophages (TAMs) utilize fatty acids to support tumor growth, while lipid metabolism products like arachidonic acid modulate immune cell functions, impacting tumor immunity^78^. Additionally, fatty acid metabolism is closely associated with various cancer signaling pathways^77^. Research on retinol metabolism in HCC has garnered significant attention in recent years. Studies have shown that the retinoic acid signaling pathway is dysregulated in various cancers, including liver cancer^79^. Additionally, other research has indicated that retinoic acid can regulate the immune system and promote antitumor immune responses. The effects of retinoic acid on TAMs and other immune cells are also under investigation, particularly regarding its potential to modulate immune cell polarization and enhance antitumor activity^80^. Currently, there is no clear report on the impact of efferocytosis-related prognostic genes (SLC26A6, TYRO3, and PDK4) on the aforementioned pathways and their primary regulatory mechanisms in HCC. Future research could focus on exploring the specific roles of these three genes in key metabolic and signaling pathways, investigating how they influence HCC progression and treatment response through the regulation of complement and coagulation cascades, drug metabolism by CYP, fatty acid metabolism, xenobiotic metabolism by CYP, and retinol metabolism.

Studies have shown that IL-33 released by the liver inhibits tumor growth by promoting the activation of CD4^+^ and CD8^+^ T cells in HCC^81^. Additionally, in the HCC mouse model, activation of adaptive immunity induced the expansion of CD4^+^ T cell populations expressing choline acetyltransferase (ChAT). Genetic ablation of the ChAT gene in CD4^+^T cells resulted in an increased prevalence of preneoplastic cells, impairing their antitumor immune function and thereby exacerbating the progression of HCC^82^. Interestingly, Yan Xu et al. discovered that inhibiting eEF2K significantly promotes NK cell proliferation and reduces apoptosis. Furthermore, combination treatment with NH125 and PD-1 inhibitors resulted in a marked increase in NK cell infiltration within HCC tumors in mouse models^83^. In this study, PDK4 and TYRO3 exhibited the highest correlation with activated CD4^+^T cells, while SLC26A6 showed the strongest association with NK cells. It is speculated that these prognostic genes may contribute to the onset and progression of HCC by influencing the infiltration of CD4^+^T cells and NK cells.

The conventional perspective posits that the process of efferocytosis, responsible for clearing dying cells within the TME, primarily exerts immunosuppressive effects^60^. However, our study reveals a more nuanced interaction. In this study, we integrated the three ERGs associated with HCC (SLC26A6, TYRO3, and PDK4) into a risk prognostic model for the first time, which proved highly effective in assessing the prognostic risk of HCC patients. Patients with high risk group exhibited significantly higher infiltration of activated CD4^+^ T cells, type 2 T helper cells (Th2 cells), and activated dendritic cells (DCs). Conversely, those with low risk group showed a marked increase in the infiltration of effector memory CD8^+^ T cells, eosinophils, γδ T cells, and natural killer cells (NKs) Activated CD4^+^ T cells, by differentiating into immunosuppressive subsets like Regulatory T cells (Tregs), may promote tumor progression and foster an immunosuppressive TME^84^. Th2 cells secrete IL-4, IL-5, and IL-13, promoting immunosuppression and tumor growth. Their increased infiltration may drive M2 macrophage polarization, enhancing the anti-inflammatory environment and advancing HCC progression^85^. Activated DCs enhance antigen presentation and T-cell activation, but mature DCs can recruit and activate Tregs in the tumor stroma, suppressing immune responses and correlating with poor patient prognosis^86^. Effector memory CD8^+^ T cells are central to antitumor immune responses, possessing the ability to rapidly recognize tumor antigens and effectively eliminate tumor cells^87^. Eosinophils play a dual role in tumor immunity by secreting cytotoxic proteins and modulating the TME^88^. γδT cells exhibit potent cytotoxicity, enabling them to directly eliminate tumor cells while simultaneously secreting cytokines that enhance antitumor immunity^89^. NK cells are essential in tumor immunity, directly killing tumor cells or inducing their apoptosis. These results suggest that, compared to the low-risk group, the immune function within the TME of the high-risk group may be significantly suppressed, thereby further promoting immune evasion by tumor cells and demonstrating a clear pro-tumor effect. With the significant progress made in immune therapy strategies such as immune checkpoint inhibitors in cancer treatment, there is an increasing focus among researchers on how to enhance the efficacy of immunotherapy through the precise targeting of immune-related genes. Our findings suggest that the ERG gene is closely linked to immune responses within the TME, indicating that ERG could serve as a potential target for the next generation of immunotherapies.

Recently, numerous scientific studies have focused on the role of immune checkpoint proteins (ICPs) in cancer progression. In this study, we found that immune checkpoint molecules such as CD27, CD70, CTLA4, CD80, CD86, ICOS, TNFRSF9, and TNFSF9 were significantly elevated in the high-risk group. Research has shown that ICPs, such as PD-1, CTLA-4, and ICOS, promote tumor growth directly or indirectly through their interactions with corresponding ligands, including PD-L1, CD80/86, and ICOS ligand^90^. The dysregulation of the CD70-CD27 axis has been associated with tumor progression and immune suppression, and has been reported in patients with hematological malignancies as well as solid tumors, such as human B cell malignancies, acute myeloid leukemia, HCC and non-small cell lung cancer^91^, ^92^. The binding of TNFRSF9 to its ligand TNFSF9 can trigger a variety of T cell responses^93^. In monocytes, crosslinking of TNFSF9 induces activation, migration, proliferation, differentiation, survival, maturation, and the production of pro-inflammatory cytokines, while inhibiting T lymphocyte proliferation and promoting their apoptosis^94^. The antitumor activity of TNFRSF9-targeted monoclonal antibodies (mAb) has been extended to various cancer models, including melanoma, lymphoma, and liver cancer^95^, ^96^. Overall, the elevated levels of immune-related inhibitory molecules in the high-risk group suggest that our prognostic gene model can effectively predict the immune status of HCC and provide potential targets for immunotherapy. This finding deepens our understanding of the role of immune checkpoint molecules in tumor immune evasion and opens new avenues for immunotherapy in HCC. Targeting these molecules holds promise for achieving more precise treatments and improving patient outcomes.

The concept of competing endogenous RNA (ceRNA) networks has gained significant attention in recent years. Studies have demonstrated that long non-coding RNAs (lncRNAs) regulate gene expression by interacting with miRNAs and mRNAs. lncRNAs are incapable of encoding proteins but can directly exert biological functions. MicroRNAs (miRNAs), a class of non-coding RNAs approximately 20-25 nucleotides in length, are known to play crucial regulatory roles in the proliferation, apoptosis, migration, and metastasis of tumor cells^97^. In this study, based on the construction of the ceRNA network, we identified 53 lncRNAs regulating the mRNAs of SLC26A6, TYRO3, and PDK4 through 21 miRNAs, such as TYRO3-hsa-miR-203b-5p-NUTM2A-AS1,SLC26A6-hsa-let-7g-5p-AC006064.5, and PDK4-hsa-miR-367-3p-XIST. Studies have demonstrated that lncRNA NUTM2A-AS1 enhances the growth, invasion, epithelial-mesenchymal transition (EMT), and stemness of HCC cells by activating the Wnt/β-catenin signaling pathway, while also inhibiting apoptosis^98^. Moreover, NUTM2A-AS1 functions as a molecular sponge for miR-186-5p, leading to the upregulation of the KLF7/Wnt/β-catenin pathway and thereby accelerating the progression of HCC^98^. Hsa-miR-203b-5p is a well-established miRNA with inhibitory effects, regulating key biological processes such as cell proliferation, migration, and invasion in tumor cells^99^. Although direct evidence for the interaction between TYRO3, hsa-miR-203b-5p, and NUTM2A-AS1 has yet to be reported, it is plausible to hypothesize that miR-203b-5p may modulate the expression of TYRO3 and NUTM2A-AS1, thereby influencing tumor-related biological processes. Consequently, future experimental studies are warranted to further elucidate this potential interaction. SLC26A6 is a solute carrier protein responsible for the transport of ions and small molecules, with its aberrant expression being associated with tumorigenesis and cancer progression^54^. Members of the let-7 family typically act as tumor suppressors, and their downregulation is closely linked to tumor malignancy^100^. AC006064.5 may regulate let-7g-5p through a "sponge" mechanism, thereby targeting SLC26A6 or its related genes to modulate tumor cell behavior. Additionally, XIST functions by relieving the inhibition of let-7, thereby modulating the paracrine IL-6 proinflammatory signaling pathway to promote cancer stem cell self-renewal^101^. Hsa-miR-367-3p is closely related to the progression, proliferation, migration, and invasion of HCC^102^. However, the exact interaction between the PDK4-hsa-miR-367-3p-XIST axis in the progression of HCC has yet to be elucidated, warranting further investigation into its underlying mechanisms in future studies.

Finally, drug sensitivity analysis identified the top five drugs correlated with the expression of prognosis genes, including BI.2536, S-Trityl-L-cysteine, GW843 682X, A-443654, and PD-173074, providing important insights for the development of new clinical therapies for HCC. BI2536, a potent inhibitor of Polo-like kinase 1 (PLK1), has demonstrated potential as an effective chemotherapeutic agent. Emerging evidence suggests that the BI.2536 may play a significant role in the clinical management of HCC patients with multidrug resistance^103^. BI2536 has also been identified as an effective therapeutic agent against ovarian cancer. It inhibits proliferation, arrests the cell cycle, and induces both apoptosis and pyroptosis, thereby suppressing tumor growth^104^. PD-173074 is an fibroblast growth factor receptor (FGFR) inhibitor that has been shown to reverse ABC transporter-mediated drug resistance in cancer cells^105^. Therefore, we hypothesize that BI.2536 and PD-173074 could serve as potential therapeutic agents for HCC, offering the prospect of overcoming drug resistance. However, further exploration of additional compounds is essential to identify other promising treatment options.

In summary, this study utilized transcriptomics data and ERGs obtained from public databases and published literature. Through a series of bioinformatics analyses, three prognostic genes—SLC26A6, TYRO3, and PDK4—were identified, and a corresponding risk model was constructed. Enrichment and drug sensitivity analyses revealed significant differences between high- and low-risk groups, providing deeper insights into the pathogenesis of HCC and offering a theoretical basis for the development of new diagnostic markers and treatment strategies. Although the AUC value of the constructed HCC risk prediction model is relatively low, it establishes a foundational framework for further research. Several limitations of this study should be acknowledged. First, the retrospective nature of public databases and published literature may introduce selection bias. Future studies can incorporate larger datasets to enhance predictive performance and assist clinicians in developing personalized treatment strategies. Additionally, the drug sensitivity (IC50) analysis and the lack of experimental validation for prognostic genes necessitate combined in vitro and in vivo experiments. Such experiments would help clarify the mechanisms underlying gene-related drug responses, refine the predictive model, and contribute to novel therapeutic development. Finally, the limited sample size in single-cell analysis may not fully capture the cellular heterogeneity of the HCC microenvironment. Prospective clinical studies will be essential to validate the model's performance in future work. Overall, this study not only identifies a set of potential therapeutic targets but also lays a solid groundwork for advancing precision medicine and innovative treatment strategies in HCC, thereby supporting progress toward personalized clinical management.

## Supplementary Material

Supplementary figures and tables.

## Figures and Tables

**Table 1:** Three HCC prognosis-related ERGs identified through multivariate Cox regression analysis.

Gene	coef	HR	HR.95L	HR.95H	pvalue
SLC26A6	0.25748	1.293666	1.031382335	1.622649	0.025930234
TYRO3	0.160245	1.173799	0.953433373	1.445096	0.130921854
PDK4	-0.0781	0.924875	0.821827824	1.040842	0.195050565

Abbreviations: coef, Coefficient; HR, Hazard Ratio; HR.95L, 95% Confidence Interval Lower Bound of HR; HR.95H, 95% Confidence Interval Upper Bound of HR.

## Data Availability

Data used in the preparation of this manuscript are available within the article and supplementary data. Further information and requests for resources and reagents should be directed to and will be promptly fulfilled by the corresponding authors.
