# Supplementary material for: The efferocytosis-related genes of SLC26A6, TYRO3, and PDK4 have been identified as predictors of prognosis in hepatocellular carcinoma and are associated with the immune status
Source: Int J Med Sci. 2026 Mar 4;23(4):1369–94. doi: 10.7150/ijms.120781 (PMC13048898; doi:10.7150/ijms.120781)
Supplement: Supplementary file 1 — Supplementary figures and tables. [file ijmsv23p1369s1.pdf]

SUPPORTING INFORMATION

SUPPLEMENTARY TABLE INFORMATION

Table S1. 74 genes in cell efferocytosis-related.

id
P2RY2
PGE2
PANX1
STAB2
ADGRB1
TIM4
TYRO3
MERTK
AXL
ELMO1
ELMO2
ELMO3
DOCK1
DOCK2
DOCK3
DOCK4
DOCK5
RAC1
CD47
SIRPA
SLC2A1
SLC16A2
ADAM17
ADAM10
DNASE1
DNASE1L1
DNASE2
PPARG
TIM3
ADAM9
MBTPS1
HIF1A
ARNT
EPO
TNF
TNFR

LRP1
ABCA1
SLC16A1
MCF2
SLC66A1
SGK1
IL10
CCL21
HMBG1
CX3CL1
CX3CR1
CXCR4
P2RY12
P2RY6
P2RX7
SLC26A6
SLC35A4
SLC46A1
SLC7A7
SLC14A1
PPARA
NR1H4
SLC25A1
SLC25A10
SLC20A1
UCP2
CPT1A
DNM1L
SLC12A4
SLC6A6
SLC4A7
WNK1
OSR1
SPAK
PDK1
PDK4
ARG1
ODC1

Table S2. Quantitative real time PCR primers.

Primers	Sequence
Forward primer for SLC26A6	GGACCCCTCTGTTATCCCCT
Reverse primer for SLC26A6	ATGTTCCTAGCACTTGGCCC
Forward primer for TYRO3	CAAGGGACCAACTGGGATCC
Reverse primer for TYRO3	TAGCTTGAGAGGTAGGCGGT
Forward primer for PDK4	GTTCACTTTGCTGCTGAGCC
Reverse primer for PDK4	CAGGATTCGGTCACACCACA
Forward primer for GAPDH	TAGGCAGCAGCAAGCATTCC
Reverse primer for GAPDH	ACGAAGCCCTTCCAGGAGAA

Table S3. Gene Set Enrichment Analysis (GSEA) of distinct risk groups within TCGA-LIHC dataset.

ID	Description	setSize	enrichmentScore	NES	pvalue	p.adjust	qvalue	rank	leading_edge	core_enrichment
KEGG_DRUG_METABOLISM_CYTOCHROME_P450	KEGG_DRUG_METABOLISM_CYTOCHROME_P450	60	-0.778207815	-2.9065331	1e-10	2.6e-09	1.849624060150038e-09	1294	tags=63%, list=9%, signal=58%	FMO5/MAOA/MAOB/GSTA2/ALDH1A3/GSTA1/UGT1A1/CYP2C18/ALDH3A1/UGT2B15/ADH6/CYP2E1/FMO4/AOX1/CYP3A5/ADH1A/FMO3/UGT1A4/CYP2C9/UGT1A3/FMO2/UGT2B10/UGT2B7/GSTZ1/GSTM5/UGT2B17/CYP3A43/ADH1C/ADH4/CYP3A4/ADH1B/CYP2C8/CYP2A6/GSTM1/CYP1A2/CYP2B6/CYP2A7/CYP2A13
KEGG_COMPLEMENT_AND_COAGULATION_CASCADES	KEGG_COMPLEMENT_AND_COAGULATION_CASCADES	68	-0.753413808	-2.8671811	1e-10	2.6e-09	1.849624060150038e-09	1954	tags=69%, list=13%, signal=60%	C4BPB/C5/C8G/PLAT/C4A/SERPINF2/C4B/A2M/CD55/SERPINA5/FGG/PROS1/PROC/FGA/F5/F7/MASP2/FGB/CFH/F2/SERPIND1/BDKRB2/C3/F8/C9/CFI/C1R/F13B/C1S/MBL2/SERPING1/THBD/MASP1/C8B/VWF/F12/KNG1/C4BPA/CPB2/F11/KLKB1/SERPINC1/C8A/PLG/C6/F9/C7
KEGG_RETINOL_METABOLISM	KEGG_RETINOL_METABOLISM	53	-0.772871066	-2.7989144	1e-10	2.6e-09	1.849624060150038e-09	920	tags=55%, list=6%, signal=52%	UGT1A1/CYP2C18/UGT2B15/ADH6/RDH5/CYP3A5/ADH1A/UGT1A4/CYP4A11/CYP2C9/UGT1A3/UGT2B10/UGT2B7/UGT2B17/RDH16/CYP4A22/CYP1A1/CYP3A43/ADH1C/ADH4/CYP3A4/ADH1B/CYP2C8/CYP2A6/CYP1A2/CYP2B6/CYP2A7/CYP26A1/CYP2A13

KEGG_FATTY_ACID_METABOLISM	KEGG_FATTY_ACID_METABOLISM	41	-0.792795532	-2.686791	1e-10	2.6e-09	1.849624060150038e-09	1705	tags=73%, list=11%, signal=65%	ACADS/HADHB/ECI2/ECHS1/ALDH9A1/ALDH1B1/ACSL5/HADH/ALDH7A1/CPT2/ACADSB/CPT1A/ACOX1/ACAT1/ACAA1/ADH6/ACADM/GCDH/ALDH2/ACSL6/ACAA2/ADH1A/ACSL1/ACADL/CYP4A11/EHHADH/CYP4A22/ADH1C/ADH4/ADH1B
KEGG_METABOLISM_OF_XENOBIOTICS_BY_CYTOCHROME_P450	KEGG_METABOLISM_OF_XENOBIOTICS_BY_CYTOCHROME_P450	57	-0.715954059	-2.638622	1e-10	2.6e-09	1.849624060150038e-09	1171	tags=53%, list=8%, signal=49%	EPHX1/GSTA2/ALDH1A3/GSTA1/UGT1A1/CYP2C18/ALDH3A1/UGT2B15/ADH6/CYP2E1/CYP3A5/ADH1A/UGT1A4/CYP2C9/UGT1A3/UGT2B10/UGT2B7/GSTZ1/GSTM5/UGT2B17/CYP1A1/CYP3A43/ADH1C/ADH4/CYP3A4/ADH1B/CYP2C8/GSTM1/CYP1A2/CYP2B6
KEGG_PEROXISOME	KEGG_PEROXISOME	78	-0.664660161	-2.596891	1e-10	2.6e-09	1.849624060150038e-09	2567	tags=56%, list=17%, signal=47%	PEX14/PAOX/PEX13/PEX7/IDH1/SOD2/NUDT12/HACL1/PEX12/PEX3/PEX11A/PEX1/CRAT/GSTK1/PHYH/HMGCL/SOD1/DHRS4/ECI2/PECR/HSD17B4/ACSL5/PIPOX/AMACR/ACOX2/ACOX1/ACAA1/PXMP2/MLYCD/PEX11G/NOS2/CAT/BAAT/AGXT/ACSL6/ACSL1/SCP2/EPHX2/SLC27A2/DAO/HAO1/EHHADH/HAO2/XDH
KEGG_TRYPTOPHAN_METABOLISM	KEGG_TRYPTOPHAN_METABOLISM	35	-0.785983549	-2.581422	1e-10	2.6e-09	1.849624060150038e-09	1272	tags=66%, list=8%, signal=60%	MAOA/ECHS1/ALDH9A1/MAOB/ALDH1B1/HADH/ALDH7A1/ACAT1/ACMSD/HAAO/GCDH/TDO2/ALDH2/CAT/AOX1/OGDHL/KMO/INMT/EHHADH/AADAT/CYP1A1/IDO2/CYP1A2

KEGG_PPAR_SIGNALING_PATHWAY	KEGG_PPAR_SIGNALING_PATHWAY	62	-0.643265107	-2.400563	2.28460777695082e-09	5.19748269256312e-08	3.69745732322304e-08	1525	tags=50%, list=10%, signal=45%	PPARA/APOA1/LPL/ACSL5/PLIN1/CYP27A1/CPT2/CPT1A/ACOX2/ACOX1/ACAA1/ANGPTL4/ACADM/APOC3/HMGCS2/ACSL6/CYP7A1/ACSL1/APOA5/SCP2/PCK2/ACADL/SLC27A2/CYP4A11/EHHADH/RXRG/PCK1/CYP4A22/SLC27A5/CYP8B1/FABP4
KEGG_VALINE_LEUCINE_AND_ISOLEUCINE_DEGRADATION	KEGG_VALINE_LEUCINE_AND_ISOLEUCINE_DEGRADATION	43	-0.71776679	-2.455081	2.70021222660455e-09	5.46042916935586e-08	3.88451583476443e-08	2447	tags=84%, list=16%, signal=70%	DLD/ALDH3A2/HMGCS1/MCCC1/ACAD8/AUH/MCCC2/ACADS/HADHB/HSD17B10/HMGCL/PCCA/PCCB/HIBCH/ECHS1/ALDH9A1/MCEE/DBT/ALDH1B1/HIBADH/HADH/ALDH7A1/BCKDHA/ACADSB/IVD/ACAT1/ACAA1/ACADM/ALDH2/HMGCS2/BCKDHB/AOX1/ACAA2/ABAT/EHHADH/ALDH6A1
KEGG_DRUG_METABOLISM_OTHER_ENZYMES	KEGG_DRUG_METABOLISM_OTHER_ENZYMES	45	-0.706956777	-2.477378	3.8476126178519e-09	7.00265496449046e-08	4.98164581048194e-08	1100	tags=49%, list=7%, signal=45%	NAT1/CES1/UGT1A1/UGT2B15/UPB1/CES2/DPYS/CYP3A5/UGT1A4/CES5A/UGT1A3/UGT2B10/UGT2B7/UGT2B17/XDH/CYP3A43/CYP3A4/NAT2/CYP2A6/UPP2/CYP2A7/CYP2A13
KEGG_GLYCINE_SERINE_AND_THREONINE_METABOLISM	KEGG_GLYCINE_SERINE_AND_THREONINE_METABOLISM	30	-0.769447596	-2.441214	2.13097665656856e-08	3.52579774086798e-07	2.5082308972051e-07	2002	tags=70%, list=13%, signal=61%	SRR/AOC3/GLDC/GCAT/MAOA/MAOB/GLYCTK/PIPOX/SHMT1/AGXT2/CBS/GATM/ALAS1/SARDH/AGXT/DAO/DMGDH/SDS/GNMT/BHMT/CTH

KEGG_CELL_CYCLE	KEGG_CELL_CYCLE	121	0.556907965490148	2.06090833599798	3.43981329721022e-08	5.2170501674355e-07	3.71137750488471e-07	1835	tags=35%, list=12%, signal=31%	CDC20/SFN/SMC1B/CDC25A/PLK1/CDKN1C/CDC6/BUB1B/PTTG1/ORC6/CCNB1/TTK/ORC1/E2F2/CDK1/CCNB2/BUB1/MCM2/CDC45/CDC7/MCM4/PKMYT1/MAD2L1/CCNE2/MCM6/CDC25C/RBL1/E2F5/CHEK1/CDKN2A/TGFB2/MCM7/YWHAZ/CDC25B/TGFB1/MCM3/ESPL1/E2F1/E2F3/MCM5/CDK4/DBF4
KEGG_STEROID_HORMONE_BIOSYNTHESIS	KEGG_STEROID_HORMONE_BIOSYNTHESIS	44	-0.689373111	-2.385796	3.98939816852018e-08	5.58515743592826e-07	3.97324675893103e-07	1178	tags=43%, list=8%, signal=40%	HSD17B8/UGT1A1/UGT2B15/CYP11A1/CYP3A5/CYP7A1/SRD5A1/UGT1A4/UGT1A3/UGT2B10/UGT2B7/HSD17B6/UGT2B17/SRD5A2/CYP1A1/CYP3A43/CYP3A4/HSD11B1/AKR1D1
KEGG_RIBOSOME	KEGG_RIBOSOME	86	0.613636109382665	2.16306907582926	4.35880977007983e-08	5.66645270110378e-07	4.0310797121791e-07	4263	tags=73%, list=28%, signal=53%	RPL8/RPL36A/RPSA/RPS19/RPL7/RPL23A/RPL38/RPS21/RPLP2/RPL18A/RPL35/RPL14/RPL9/RPS24/RPS2/RPL28/RPL37A/RPL32/RPL27A/RPS3/RPL37/RPS7/RPL35A/RPS5/RPS20/RPL39/RPS10/RPS27A/RPLP0/RPS16/RPL18/MRPL13/RPL22L1/RPL30/RPS8/RPL36/RPL13/RPL7A/RPL6/RPL27/RPS15/RPL23/RPL17/RPL5/RPL29/RPL13A/RPS11/RPL24/RPLP1/RPS12/RPS18/RPS27/RPS3A/RPL12/RPS6/RPL19/RPS23/RPS17/RPL4/RPL31/RPS4X/RPS28/RPS9
KEGG_PROPANOATE_METABOLISM	KEGG_PROPANOATE_METABOLISM	30	-0.749884788	-2.379147	1.2080484074065e-07	1.46576540097945e-06	1.04273652007214e-06	1436	tags=67%, list=9%, signal=60%	PCCA/PCCB/ACSS3/HIBCH/ECHS1/ALDH9A1/MCEE/SUCLG2/ACACB/ALDH1B1/ALDH7A1/SUCLG2P2/ACAT1/MLYCD/LDHC/ACADM/ALDH2/ABAT/EHHADH/ALDH6A1

KEGG_HISTIDINE_METABOLISM	KEGG_HISTIDINE_METABOLISM	27	-0.756999719	-2.36316	1.77318773735176e-07	1.89835393057659e-06	1.35047734795212e-06	1592	tags=63%, list=11%, signal=56%	HNMT/MAOA/ACY3/ALDH9A1/MAOB/HAL/ALDH1B1/ALDH1A3/ALDH7A1/ALDH3A1/FTCD/ASPA/ALDH2/HDC/AMDHD1/UROC1/CNDP1
KEGG_ARGININE_AND_PROLINE_METABOLISM	KEGG_ARGININE_AND_PROLINE_METABOLISM	49	-0.646530447	-2.302563	1.70026556077031e-07	1.89835393057659e-06	1.35047734795212e-06	1637	tags=57%, list=11%, signal=51%	AGMAT/ALDH4A1/LAP3/SAT1/ACY1/MAOA/ALDH9A1/NAGS/PRODH2/MAOB/ALDH1B1/GLUL/ALDH7A1/ARG2/ASL/GOT1/GOT2/GATM/NOS2/ASS1/ALDH2/OAT/OTC/CPS1/ARG1/DAO/PRODH/GLS2
KEGG_PRIMARY_BILE_ACID_BIOSYNTHESIS	KEGG_PRIMARY_BILE_ACID_BIOSYNTHESIS	16	-0.862687824	-2.355046	1.92184366682258e-07	1.94319748534283e-06	1.38237877788993e-06	1845	tags=88%, list=12%, signal=77%	CYP7B1/CYP46A1/CH25H/HSD17B4/CYP27A1/AMACR/ACOX2/BAAT/CYP7A1/SCP2/CYP39A1/SLC27A5/AKR1D1/CYP8B1
KEGG_TYROSINE_METABOLISM	KEGG_TYROSINE_METABOLISM	32	-0.68671918	-2.200384	3.55661226677359e-06	3.40686017133049e-05	2.42361943940804e-05	1272	tags=59%, list=8%, signal=54%	FAH/AOC3/MAOA/MAOB/ALDH1A3/GOT1/ALDH3A1/GOT2/ADH6/HGD/AOX1/ADH1A/HPD/DBH/GSTZ1/ADH1C/ADH4/ADH1B/TAT

KEGG_BUTANOATE_METABOLISM	KEGG_BUTANOATE_METABOLISM	31	-0.664840923	-2.109575	2.86454637406425e-05	0.000260673720039847	0.000185441686321002	1705	tags=65%, list=11%, signal=57%	ACADS/HMGCL/BDH2/ECHS1/L2HGDH/ALDH9A1/ALDH1B1/BDH1/HADH/ALDH7A1/ACSM1/ALDH5A1/ACAT1/ALDH2/HMGCS2/ACSM3/ABAT/EHHADH/ACSM5/ACSM2A
KEGG_LYSINE_DEGRADATION	KEGG_LYSINE_DEGRADATION	39	-0.609227872	-2.047904	4.20500811767143e-05	0.000364434036864858	0.000259256139585758	1568	tags=38%, list=10%, signal=35%	SETD7/ECHS1/ALDH9A1/ALDH1B1/HADH/ALDH7A1/PIPOX/ACAT1/GCDH/ALDH2/OGDHL/EHHADH/AADAT/BBOX1/AASS
KEGG_CITRATE_CYCLE_TCA_CYCLE	KEGG_CITRATE_CYCLE_TCA_CYCLE	30	-0.642362237	-2.038012	8.32623423813699e-05	0.000688806650609514	0.000490012828368827	3778	tags=73%, list=25%, signal=55%	SDHC/IDH2/OGDH/SUCLG1/IDH3A/ACO2/SUCLA2/MDH1/SDHA/DLD/IDH1/DLST/SDHB/FH/SDHD/SUCLG2/PC/ACO1/SUCLG2P2/OGDHL/PCK2/PCK1
KEGG_CYSTEINE_AND_METHIONINE_METABOLISM	KEGG_CYSTEINE_AND_METHIONINE_METABOLISM	32	-0.624129985	-1.999836	0.000100779145906376	0.000795850709942172	0.000566163315921846	979	tags=38%, list=6%, signal=35%	ADI1/GOT1/GOT2/LDHC/CBS/MAT1A/CDO1/SDS/DNMT3L/BHMT/CTH/TAT

KEGG_GLYCOLYSIS_GLUCONEOGENESIS	KEGG_GLYCOLYSIS_GLUCONEOGENESIS	51	-0.535964931	-1.915906	0.0001049473463666001	0.0007958507099420172	0.0005661633159921846	1260	tags=41%, list=8%, signal=38%	ALDH9A1/ENO3/ALDH1B1/ALDH1A3/PGM2/PGM1/ALDH7A1/PKLR/ALDH3A1/ADH6/LDHC/ALDH2/ADH1A/PCK2/ALDOB/FBP1/PCK1/ADH1C/ADH4/ADH1B/GCK
KEGG_PORPHYRIN_AND_CHLOROPHYLL_METABOLISM	KEGG_PORPHYRIN_AND_CHLOROPHYLL_METABOLISM	32	-0.618847492	-1.98291	0.000129345937698214	0.000941638426442998	0.000669875803658119	960	tags=31%, list=6%, signal=29%	CP/UGT1A1/ALAD/UGT2B15/ALAS1/UGT1A4/UGT1A3/UGT2B10/UGT2B7/UGT2B17
KEGG_STARCH_AND_SUCROSE_METABOLISM	KEGG_STARCH_AND_SUCROSE_METABOLISM	39	-0.570099511	-1.916375	0.0002183973505010768	0.00152878145351237	0.00108756575351083	1370	tags=46%, list=9%, signal=42%	AMY2B/PYGL/ENPP1/PGM2/UGT1A1/PGM1/UGT2B15/AGL/UGP2/UGT1A4/UGT1A3/UGT2B10/UGT2B7/UGT2B17/GBA3/TREH/GYS2/GCK
KEGG_BETA_ALANINE_METABOLISM	KEGG_BETA_ALANINE_METABOLISM	21	-0.681188106	-1.990604	0.0003212200737520705	0.0021652612378886	0.0015403535700421	1351	tags=67%, list=9%, signal=61%	AOC3/HIBCH/ECHS1/ALDH9A1/ALDH1B1/ALDH7A1/MLYCD/ACADM/UPB1/DPYS/ALDH2/ABAT/EHHADH/CNDP1

KEGG_ADIPOCYTOKINE_SIGNALING_PATHWAY	KEGG_ADIPOCYTOKINE_SIGNALING_PATHWAY	59	-0.492140283	-1.829644	0.000376150810386321	0.00244498026751109	0.001739343972839	3215	tags=42%, list=21%, signal=34%	PRKAB2/POMC/AKT1/TNFRSF1A/CD36/TNFRSF1B/SOCS3/RXRA/ADIPOR2/NFKBIA/PPARA/IRS1/SLC2A4/ACACB/ACSL5/CPT1A/PRKAG2/IRS2/LEPR/ACSL6/PPARGC1A/ACSL1/PCK2/RXRG/PCK1
KEGG_DNA_REPLICATION	KEGG_DNA_REPLICATION	35	0.613594669615918	1.88166731816763	0.000456150387799793	0.00286273691653663	0.00203653349181033	2874	tags=54%, list=19%, signal=44%	MCM2/MCM4/MCM6/RFC4/MCM7/DNA2/MCM3/POLD1/FEN1/MCM5/POLA2/RNASEH2A/PRIM1/POLA1/LIG1/PCNA/PRIM2/RFC2/POLD3
KEGG_LINOLEIC_ACID_METABOLISM	KEGG_LINOLEIC_ACID_METABOLISM	21	-0.667650619	-1.951044	0.000585395113486969	0.00355139702182094	0.00252644206873323	1220	tags=52%, list=8%, signal=48%	PLA2G2A/PLA2G5/CYP2C18/CYP2J2/CYP2E1/CYP3A5/CYP2C9/CYP3A43/CYP3A4/CYP2C8/CYP1A2
KEGG_PENTOSE_AND_GLUCURONATE_INTERCONVERSIONS	KEGG_PENTOSE_AND_GLUCURONATE_INTERCONVERSIONS	21	-0.665078133	-1.943527	0.00063354543710859	0.00371952482431495	0.00264604715668443	920	tags=48%, list=6%, signal=45%	UGT1A1/CRYL1/UGT2B15/UGP2/UGT1A4/UGT1A3/UGT2B10/UGT2B7/DCXR/UGT2B17

KEGG_CARDIAC_MUSCLE_CONTRACTION	KEGG_CARDIAC_MUSCLE_CONTRACTION	54	0.538917378981075	1.77988828444729	0.000733727933578244	0.00417307762222626	0.00296870183651724	3009	tags=35%, list=20%, signal=28%	TNNC1/TNNT2/CACNG4/FXYD2/MYL3/TPM2/CACNB1/CACNB3/ATP1A3/ATP1B3/CACNA2D2/ATP1A1/CACNA1D/TPM4/SLC9A1/CACNA2D4/COX6B1/CYC1/TPM3
KEGG_INSULIN_SIGNALING_PATHWAY	KEGG_INSULIN_SIGNALING_PATHWAY	126	-0.378746026	-1.582228	0.000874015238054916	0.00482032646442408	0.00342915069476091	1423	tags=15%, list=9%, signal=14%	IRS1/PYGL/SLC2A4/ACACB/PDE3B/PKLR/PPP1R3B/PRKAG2/IRS2/PIK3R1/PPARGC1A/PCK2/FOXO1/SOCS2/FBP1/PCK1/GYS2/GCK/SHC4
KEGG_PYRUVATE_METABOLISM	KEGG_PYRUVATE_METABOLISM	37	-0.560895311	-1.870152	0.00103436548063305	0.00553689757280044	0.00393891498816919	1260	tags=43%, list=8%, signal=40%	ALDH9A1/ACACB/ACYP2/ALDH1B1/PC/ALDH7A1/PKLR/HAGH/ACAT1/LDHC/GRHPR/ALDH2/LDHD/PCK2/ACOT12/PCK1
KEGG_ASCORBATE_AND_ALDARATE_METABOLISM	KEGG_ASCORBATE_AND_ALDARATE_METABOLISM	19	-0.666362686	-1.894143	0.00140034615695799	0.00728180001618154	0.00518022788889722	1260	tags=58%, list=8%, signal=53%	ALDH9A1/ALDH1B1/UGT1A1/ALDH7A1/UGT2B15/ALDH2/UGT1A4/UGT1A3/UGT2B10/UGT2B7/UGT2B17

KEGG_SPLICEOSOME	KEGG_SPLICEOSOME	124	0.426913336331597	1.58493916838579	0.00180233100665709	0.00911178453365527	0.00648206765552111	6009	tags=56%, list=40%, signal=34%	SNRPB/SNRPD1/ALYREF/SNRPD2/SF3B4/SNRPA/PPIH/SNRPF/LSM4/SNRPE/SNRPA1/LSM7/PUF60/SF3A2/PPIL1/LSM2/ISY1/NCBP2/PRPF3/SNRPC/EFTUD2/TCERG1/LSM8/SNRNP40/SNRPG/THOC1/PRPF31/U2AF2/TXNL4A/PQBP1/RBM17/PRPF40B/PRPF6/U2SURP/THOC3/CCDC12/SNRPB2/PPIE/SRSF9/HNRNPA3/HSPA6/HNRNPA1/HNRNPA1L2/SNRNP70/ACIN1/RBMX/HNRNPU/RBM8A/PRPF38A/BUD31/USP39/SF3A3/PHF5A/MAGOH/EIF4A3/SNRPD3/SF3B5/SRSF1/HNRNPM/HSPA2/SRSF3/PRPF4/DHX15/DDX39B/SRSF2/DDX23/MAGOHB/THOC2/PRPF38B/PRPF19
KEGG_RENIN_ANGIOTENSIN_SYSTEM	KEGG_RENIN_ANGIOTENSIN_SYSTEM	14	-0.7000752556	-1.8367288	0.0019547770961599	0.00949916682536946	0.0067576490429175	1009	tags=29%, list=7%, signal=27%	MME/ENPEP/ACE2/REN
KEGG_ABC_TRANSPORTERS	KEGG_ABC_TRANSPORTERS	39	-0.5089791733	-1.71092	0.0019833425239782	0.00949916682536946	0.0067576490429175	956	tags=28%, list=6%, signal=26%	ABCG5/ABCC6/ABCG8/ABCC11/ABCG2/ABCA9/ABCA6/ABCC9/ABCB11/ABCB4/ABCA8
KEGG_ALANINE_ASPARTATE_AND_GLUTAMATE_METABOLISM	KEGG_ALANINE_ASPARTATE_AND_GLUTAMATE_METABOLISM	29	-0.5412840922	-1.7033333	0.00346462029880832	0.0157640223595779	0.0112144288619322	1262	tags=55%, list=8%, signal=51%	ALDH4A1/ACY3/GLUL/ASL/GOT1/ALDH5A1/AGXT2/GPT2/GOT2/GPT/ASPA/ASS1/AGXT/CPS1/ABAT/GLS2

KEGG_ARACHIDONIC_ACID_METABOLISM	KEGG_ARACHIDONIC_ACID_METABOLISM	46	-0.469259255	-1.650866	0.00344205724601783	0.0157640223595779	0.0112144288619322	1320	tags=39%, list=9%, signal=36%	GGT5/PTGS2/PLA2G2A/CYP2U1/CBR1/PLA2G5/PTGIS/CYP2C18/CYP2J2/CYP4F3/CYP2E1/EPHX2/CYP4A11/CYP2C9/CYP4F2/CYP4A22/CYP2C8/CYP2B6
KEGG_HOMOLOGOUS_RECOMBINATION	KEGG_HOMOLOGOUS_RECOMBINATION	25	0.632083394501578	1.7815659656732	0.00375155364386538	0.0166532381264268	0.0118470115069433	2874	tags=44%, list=19%, signal=36%	RAD54L/EME1/XRCC2/RAD51/BLM/RAD54B/POLD1/XRCC3/BRCA2/RAD51D/POLD3
KEGG_NEUROACTIVE_LIGAND_RECEPTOR_INTERACTION	KEGG_NEUROACTIVE_LIGAND_RECEPTOR_INTERACTION	130	-0.340689378	-1.416922	0.00552798297125429	0.0239545928754352	0.0170411505128892	1598	tags=26%, list=11%, signal=24%	GRID1/NR3C1/P2RY14/PTGDR/SSTR1/PTH2R/APLNR/F2/ADRB1/BDKRB2/ADRA2B/S1PR1/CALCRL/P2RX3/GRM2/EDNRB/ADRB2/PTH1R/F2RL3/LEPR/GRIN2B/CHRNA4/ADRA1B/PLG/GRPR/NPY1R/AVPR1A/GHR/HTR2B/GABBR2/VIPR1/GCGR/DRD1/ADRA1A
KEGG_PRION_DISEASES	KEGG_PRION_DISEASES	33	-0.511251514	-1.650875	0.00704191966851251	0.029805344109134	0.021203321720205	1917	tags=36%, list=13%, signal=32%	C5/C8G/IL6/SOD1/FYN/C9/EGR1/NCAM2/C8B/C8A/C6/C7

KEGG_NOD_LIKE_RECEPTOR_SIGNALING_PATHWAY	KEGG_NOD_LIKE_RECEPTOR_SIGNALING_PATHWAY	56	0.481106518689453	1.59906767270687	0.00750002657534801	0.0310228371980304	0.0220694561906174	3675	tags=32%, list=24%, signal=24%	CXCL1/MAPK13/PYCARD/CXCL8/IL18/MAPK12/TNF/RIPK2/CCL13/IL1B/CARD9/BIRC3/PSTPIP1/HSP90AB1/CCL8/MAPK3/MAPK10/NOD2
KEGG_BIOSYNTHESIS_OF_UNSATURATED_FATTY_ACIDS	KEGG_BIOSYNTHESIS_OF_UNSATURATED_FATTY_ACIDS	20	-0.583939905	-1.677297	0.00972788668531153	0.0384885951462326	0.0273805506245611	3009	tags=60%, list=20%, signal=48%	HSD17B12/HADHA/ELOVL6/ELOVL5/ELOVL2/ACOT4/ACOT1/ACOT2/PECR/ACOX1/ACAA1/BAAT
KEGG_GLYCOSPHINGOLIPID_BIOSYNTHESIS_LACTO_AND_NEOLACTO_SERIES	KEGG_GLYCOSPHINGOLIPID_BIOSYNTHESIS_LACTO_AND_NEOLACTO_SERIES	22	0.594975893348256	1.63691643545756	0.00969096445153556	0.0384885951462326	0.0273805506245611	1340	tags=41%, list=9%, signal=37%	FUT3/B3GALT2/B3GNT3/FUT2/FUT4/FUT7/B3GNT4/FUT1/ST8SIA1
KEGG_ETHER_LIPID_METABOLISM	KEGG_ETHER_LIPID_METABOLISM	23	0.571883660920913	1.57958850180632	0.0114750440401107	0.0444352769212796	0.0316109835819398	1128	tags=30%, list=7%, signal=28%	PAFAH1B3/LPCAT1/ENPP6/PLA2G2D/PLA2G7/PLA2G1B/LPCAT4

Table S4. Hallmark analyzed a table of 18 channels.

ID	Description	setSize	enrichmentScore	NES	pvalue	p.adjust	qvalue	rank	leading_edge	core_enrichment
HALLMARK_BILE_ACID_METABOLISM	HALLMARK_BILE_ACID_METABOLISM	109	-0.667840078	-2.768347983	1e-10	1e-09	5.47368421052632e-10	2397	tags=63%, list=16%, signal=54%	PAOX/PEX13/PEX7/IDH1/RXRA/FDXR/NUDT12/HACL1/PNPLA8/PEX12/ALDH1A1/ISOC1/CYP7B1/HSD17B11/LIPE/RETSAT/PEX11A/PEX1/DIO1/GSTK1/PHYH/SOD1/CYP46A1/APOA1/NEDD4/CH25H/SLC23A2/PECR/ALDH9A1/GC/HSD17B4/ACSL5/NR3C2/SERPINA6/PIPOX/CYP27A1/LONP2/AMACR/ABCG8/PXMP2/MLYCD/SLCO1A2/PEX11G/ABCA9/CAT/AGXT/AR/CYP7A1/ACSL1/ABCA6/SCP2/EPHX2/SLC27A2/AQP9/ALDH8A1/HAO1/NR1I2/TTR/HSD17B6/RXRG/GNMT/SULT1B1/BBOX1/DIO2/CYP39A1/ABCA8/SLC27A5/AKR1D1/CYP8B1
HALLMARK_XENOBIOTIC_METABOLISM	HALLMARK_XENOBIOTIC_METABOLISM	191	-0.606663402	-2.693687494	1e-10	1e-09	5.47368421052632e-10	1866	tags=52%, list=12%, signal=46%	FAH/PTGR1/GCLC/RETSAT/MCCC2/VTN/HNF4A/CDA/ID2/TMBIM6/ETS2/IGFBP4/PDLIM5/SAR1B/PROS1/BPHL/MAOA/TMEM176B/ABHD6/ALDH9A1/CA2/PAPSS2/EPHX1/CBR1/ITIH1/ANGPTL3/PC/CES1/SLC35D1/CYFIP2/GCKR/ACSM1/LPIN2/ARG2/ASL/SERPINA6/TTPA/CYP27A1/LEAP2/IGF1/POR/ENTPD5/CYP2C18/CSAD/ACOX2/ACOX1/CYP2J2/ALDH3A1/GCH1/PEMT/PINK1/FAS/PGRMC1/NDRG2/MBL2/UPB1/DHRS1/CYB5A/ALAS1/CYP2E1/ATOH8/HGFAC/RBP4/TDO2/ETFDH/ALDH2/LCAT/CAT/ENPEP/GABARAPL1/AOX1/CDO1/IGFBP1/F11/FETUB/FMO3/SLC6A12/ARG

										1/HRG/AQP9/SLC46A3/MTHFD1/PLG/DCXR/MT2A/GNMT/CYP4F2/FBP1/XDH/CYP1A1/ADH1C/HSD11B1/CCL25/CYP1A2/ESR1/TAT/PDK4/SLC22A1/CYP26A1
HALLMARK_FATTY_ACID_METABOLISM	HALLMARK_FATTY_ACID_METABOLISM	146	-0.605286649	-2.617159667	1e-10	1e-09	5.47368421052632e-10	2533	tags=56%, list=17%, signal=47%	PCBD1/PTS/SDHA/ELOVL5/DLD/IDH1/RDH11/ALDH3A2/HMGCS1/ADIPOR2/DLST/ALDH1A1/DECR1/TP53INP2/AUH/CRYZ/HSD17B11/AQP7/RETSAT/AOC3/FH/ACADS/HADHB/CRAT/HSDL2/HSD17B10/REEP6/PPARA/HMGCL/SERINC1/ACOT2/ECI2/HIBCH/BPHL/MAOA/ECHS1/ALDH9A1/MCEE/HPGD/G0S2/SDHD/CA2/EPHX1/SUCLG2/CBR1/ENO3/HSD17B4/ACSL5/GLUL/HADH/CPT2/CPT1A/ACOX1/ALDH3A1/ALAD/ACAA1/CD1D/MLYCD/ACADM/GCDH/TDO2/ETFDH/GRHPR/HMGCS2/BCKDHB/GABARAPL1/ACAA2/ACSM3/ACSL1/ACADL/INMT/CYP4A11/EHHADH/GSTZ1/GPD1/HAO2/AADAT/RDH16/CYP4A22/CYP1A1/ADH1C/CA4
HALLMARK_E2F_TARGETS	HALLMARK_E2F_TARGETS	195	0.648449976296386	2.53686379570262	1e-10	1e-09	5.47368421052632e-10	3455	tags=58%, list=23%, signal=46%	MYBL2/CDC20/KIF2C/TRIP13/AURKB/CDC25A/CENPM/PLK1/KIF18B/BIRC5/HMGA1/BUB1B/KIF4A/PTTG1/CDCA3/ORC6/GINS1/TOP2A/CDK1/CDCA8/MKI67/DLGAP5/CCNB2/ASF1B/SPC25/TACC3/CENPE/UBE2T/UBE2S/MCM2/HELLS/DEPDC1/MELK/CDKN3/CKS2/MCM4/PSMC3IP/MAD2L1/DSCC1/RACGAP1/EZH2/LMNB1/MCM6/NCAPD2/KPNA2/RRM2/PLK4/CHEK1/SPC24/TCF19/NOP56/BARD1/CDKN2A/GINS4/MCM7/SNRPB/STMN1/CIT/RAD51AP1/CDC25B/MCM3/ESPL1/TK1/NME1/POLD1/HMMR/NAP1L1/E2F8/DDX39A/SPAG5/MXD3/BRCA1/MCM5/CDK4/POLA2/TUBG1/DNMT1/MMS22L/HMGB2/RNASEH2A/TUBB/MSH2/AURKA/LIG1/RAN/CCNE1/BRCA2/SMC4/CKS1B/PCNA/PRKDC/GINS3/PA2G4/TIPIN/CSE1L/CBX5/PRIM2/RFC2/POLD3/DONSON/RAD21/ATAD2/

										TIMELESS/NASP/MTHFD2/SLBP/ILF3/LYAR/NUP107/USP1/DCTPP1/POP7/EED/LBR
HALLMARK_G2M_CHECKPOINT	HALLMARK_G2M_CHECKPOINT	187	0.65114884760462	2.52803956999726	1e-10	1e-09	5.47368421052632e-10	2431	tags=47%, list=16%, signal=40%	EFNA5/MYBL2/CDC20/UBE2C/KIF2C/AURKB/CDC25A/CENPA/PLK1/BIRC5/CDC6/RAD54L/TROAP/HMGA1/KIF23/KIF4A/PTTG1/ORC6/CENPF/KIF15/TTK/TOP2A/SLC38A1/E2F2/CDK1/MKI67/NEK2/CCNB2/BUB1/TPX2/TRAIP/EXO1/KIF11/TACC3/CENPE/NDC80/UBE2S/MCM2/POLQ/CDC45/CDC7/CCNF/CDKN3/CKS2/STIL/MAD2L1/RACGAP1/PBK/EZH2/LMNB1/MCM6/KPNA2/SLC7A1/PRC1/UCK2/PLK4/RBL1/CHEK1/BARD1/NUSAP1/STMN1/DTYMK/MARCKS/CDC25B/TGFB1/MCM3/ESPL1/E2F1/E2F3/HMMR/SNRPD1/DDX39A/MCM5/CDK4/POLA2/DBF4/KIF20B/FBXO5/GINS2/CBX1/AURKA/MTF2/BRCA2/INCENP/MEIS2/SMC4/CKS1B/SLC7A5
HALLMARK_COAGULATION	HALLMARK_COAGULATION	128	-0.494393939396	-2.09912315252	1.60179448999975e-07	1.33482874166645e-06	7.30643100701638e-07	1905	tags=43%, list=13%, signal=38%	APOC1/C8G/PLAT/RAPGEF3/A2M/GDA/PECAM1/HNF4A/CLU/FBN1/APOA1/FGG/HPN/MST1/DPP4/PROS1/PROC/TF/FGA/TMPRSS6/MASP2/ITIH1/CFH/F2/ITGB3/C3/FYN/F8/C9/ACOX2/CFI/CTSO/C1R/ANG/RGN/F13B/C1S/MBL2/SERPING1/THBD/TIMP3/C8B/VWF/F12/APOC3/HMGCS2/CPB2/F11/KLKB1/HRG/PROZ/SERPINC1/C8A/PLG/F9
HALLMARK_OXIDATIVE_PHOSPHORYLATION	HALLMARK_OXIDATIVE_PHOSPHORYLATION	183	-0.43062777	-1.88668686178	3.63700436188053e-07	2.59786025848095e-06	1.42198666780478e-06	3430	tags=33%, list=23%, signal=26%	GLUD1/NDUFB6/SUCLG1/UQCR11/AFG3L2/IDH3A/ACO2/ECI1/ISCA1/SUCLA2/NDUFS7/MDH1/SURF1/HADHA/SLC25A4/ECH1/ABCB7/AIFM1/OPA1/ACADVL/SDHA/MRPL34/DLD/PRDX3/IDH1/NDUFS1/FXN/DLST/DECR1/UQCRC2/RETSAT/NDUFA5/SDHB/FH/HADHB/PHYH/HSD17B10/MPC1/BDH2/SLC25A20/ECHS1/FDX1/ETFA/NNT/SDHD/MAOB/BCKDHA/ACADSB/POR/CPT1A/ACA

										T1/ACAA1/GOT2/ACADM/CYB5A/ALAS1/ETFDH/OAT/ACAA2/ALDH6A1/PDK4
HALLMARK_ADIPOGENESIS	HALLMARK_ADIPOGENESIS	184	-0.427238522	-1.877581528	4.43495365756476e-07	2.77184603597797e-06	1.51722098811426e-06	2866	tags=41%, list=19%, signal=34%	ECH1/PPM1B/CD36/ABCA1/RNF11/AIFM1/ITSN1/DDT/ELOVL6/STOM/PEX14/GBE1/DLD/PRDX3/GHITM/RREB1/IDH1/COQ9/MCCC1/RMDN3/ADIPOR2/GPAM/SOWAHC/SORBS1/DECR1/NKIRAS1/FAH/LIPE/RETSAT/NDUFA5/ITGA7/SDHB/ACADS/CRAT/PHYH/REEP6/ORM1/SOD1/IFNGR1/SLC5A6/FZD4/LPL/HIBCH/ECHS1/DNAJB9/TOB1/TST/SULT1A1/DBT/QDPR/COL15A1/C3/PGM1/HADH/BCKDHA/CPT2/POR/CMBL/LIFR/ACOX1/CD302/PEMT/GPHN/ANGPTL4/ACADM/MYLK/ALDH2/CAT/GADD45A/ACAA2/SCP2/SPARCL1/EPHX2/ACADL/OMD/FABP4
HALLMARK_MITOTIC_SPINDLE	HALLMARK_MITOTIC_SPINDLE	198	0.478679257673328	1.87645134141769	5.6042438412728e-07	3.11346880070711e-06	1.70421450143968e-06	2380	tags=28%, list=16%, signal=24%	KIF2C/PLK1/BIRC5/ANLN/KIF3C/KIF23/KIF4A/PIF1/CENPF/KIF15/TTK/TOP2A/CDK1/NEK2/DLGAP5/CCNB2/BUB1/TPX2/KIF11/ARHGEF2/CENPE/NDC80/ECT2/KNTC1/RHOF/RACGAP1/LMNB1/PRC1/NCK2/NUSAP1/ARHGAP4/MARCKS/FSCN1/ESPL1/CEP131/MID1IP1/CLIP2/CEP72/WASF1/PLEKHG2/CENPJ/KIF20B/ARHGEF3/SAC3D1/TUBA4A/FBXO5/FGD6/ABR/MAPRE1/CDC42EP1/SASS6/AURKA/FLNA/BRCA2/INCENP/SMC4
HALLMARK_PEROXISOME	HALLMARK_PEROXISOME	99	-0.504088586	-2.052601381	1.29088753876461e-06	6.30905827136743e-06	3.45337926432743e-06	2644	tags=45%, list=18%, signal=38%	FIS1/PEX14/ELOVL5/DHRS3/PEX13/IDH1/RDH11/SOD2/ALDH1A1/ABCB1/ISOC1/STS/HSD17B11/RETSAT/CTPS1/PEX11A/DIO1/CRAT/GSTK1/HMGCL/SOD1/ECI2/SLC23A2/ALDH9A1/CADM1/HSD17B4/ALB/ACSL5/SERPINA6/LONP2/ACOX1/ACAA1/MLYCD/CAT/ACSL1/SCP2/EPHX2/SLC27A2/NR1I2/EHHADH/TTR/ABCB4/RXRG/UGT2B17/HAO2

HALLMARK_MYC_TARGETS_V1	HALLMARK_MYC_TARGETS_V1	192	0.468548217057925	1.82996482570939	1.38799281970083e-06	6.30905827136743e-06	3.45337926432743e-06	5309	tags=56%, list=35%, signal=37%	CDC20/MCM2/CDC45/MCM4/MAD2L1/MCM6/KPNA2/TYMS/NOP56/RFC4/MCM7/NME1/FBL/NAP1L1/SNRPD1/MCM5/SNRPD2/CDK4/PABPC1/CAD/SNRPA/TRIM28/SRM/CCT5/RPL14/RAN/IMPDH2/CCT3/RPS2/EIF3B/RPS3/PCNA/NPM1/SNRPA1/RPS5/LSM7/PA2G4/RPS10/ODC1/TFDP1/CSTF2/RPLP0/CDK2/ILF2/RPL18/CCT2/HSP90AB1/HDDC2/CYC1/SMARCC1/LSM2/RRP9/CBX3/HDAC2/NCBP2/UBA2/RPL6/XPOT/EIF2S2/KPNB1/USP1/CCNA2/PPIA/PPM1G/UBE2E1/HSPE1/GNL3/RANBP1/SNRPG/SRPK1/MRPL9/UBE2L3/PRPF31/TXNL4A/PSMB3/RNPS1/SSB/RPS6/EIF3D/PSMD14/PABPC4/EXOSC7/RRM1/RUVBL2/SNRPB2/XRCC6/MRPL23/IFRD1/YWHAQ/HNRNPA3/HNRNPA1/CCT7/SET/VDAC1/BUB3/HNRNPU/EEF1B2/NOP16/PSMC4/COPS5/HSPD1/PGK1/CCT4/SNRPD3/PTGES3/HNRNPD/SRSF1/CANX
HALLMARK_ESTROGEN_RESPONSE_LATE	HALLMARK_ESTROGEN_RESPONSE_LATE	177	0.420141560703222	1.62870998591176	0.000390208222985234	0.00162586759577181	0.000889948578738254	1202	tags=19%, list=8%, signal=18%	AGR2/PKP3/KRT19/CLIC3/LAMC2/PTGES/S100A9/IGSF1/TNNC1/TFF3/CDC20/CYP26B1/SFN/GJB3/CDC6/TMPRSS3/MAPK13/KIF20A/TOP2A/TPBG/MDK/MYB/NBL1/SULT2B1/FGFR3/HSPB8/HR/FABP5/STIL/ALDH3B1/ST14/ABCA3/PLK4/CD44
HALLMARK_ANDROGEN_RESPONSE	HALLMARK_ANDROGEN_RESPONSE	92	-0.417661043	-1.674842081	0.00141483220	0.00544166234	0.00297859412	2687	tags=42%, list=18%,	LMAN1/CCND1/ELOVL5/ANKH/HMGCS1/HSD17B14/MAF/UAP1/NKX3-1/NCOA4/SEC24D/PTPN21/MAP7/B2M/MERTK/TMPRSS2/HERC3/SAT1/PTK2B/PDLIM5/FKBP5/SLC38A2/TSC22D1/DNAJB9/ELL2/

					999489	613418	630503		signal=35%	SGK1/HPGD/IQGAP2/ALDH1A3/ABHD2/ADAMTS1/HOMER2/LIFR/STEAP4/CDC14B/SORD/AKAP12/INSIG1/AZGP1
HALLMARK_GLYCOLYSIS	HALLMARK_GLYCOLYSIS	188	0.390509291560107	1.51795750037435	0.00245605935050394	0.00877164053751407	0.00480131903106033	2231	tags=27%, list=15%, signal=23%	HS6ST2/PPFIA4/G6PD/SLC16A3/PKM/GAL3ST1/TFF3/EGLN3/GPC4/PFKP/HK2/MIOX/CENPA/ENO2/KIF20A/QSOX1/B3GNT3/SOX9/TPBG/CDK1/ADORA2B/VLDLR/ARTN/EFNA3/GPC3/STC2/ALDOA/DEPDC1/DPYSL4/TGFA/CHST1/IER3/CD44/STMN1/DSC2/HOMER1/GNPDA1/ENO1/HMMR/ELF3/SPAG4/PYGB/AGRN/BIK/PLOD2/AURKA/IGFBP3/RBCK1/RRAGD/MIF
HALLMARK_INTERFERON_ALPHA_RESPONSE	HALLMARK_INTERFERON_ALPHA_RESPONSE	95	-0.383802096	-1.547202475	0.00775307795593527	0.0242283686122977	0.0132618438719945	3080	tags=38%, list=20%, signal=30%	TRIM5/PARP14/ELF1/UBE2L6/OAS1/RTP4/TRIM26/HLA-C/BST2/IRF2/UBA7/IFIT2/TMEM140/HELZ2/GMPR/IFIH1/PARP9/EPSTI1/IFIT3/USP18/DHX58/IFITM1/B2M/ISG15/MX1/LAP3/RSAD2/IFI44/CMPK2/DDX60/IFI44L/HERC6/OASL/TXNIP/C1S/IFI27
HALLMARK_INTERFERON_GAMMA_RESPONSE	HALLMARK_INTERFERON_GAMMA_RESPONSE	193	-0.314887147	-1.397024933	0.00756256644982824	0.0242283686122977	0.0132618438719945	2746	tags=28%, list=18%, signal=23%	SOCS3/CD40/BST2/IRF2/IFIT2/SPPL2A/IRF4/HELZ2/ITGB7/CFB/MYD88/IFIH1/SOD2/IRF8/EPSTI1/LATS2/IFIT3/NFKBIA/ISOC1/PDE4B/IL6/TNFSF10/USP18/DHX58/B2M/ISG15/MX1/LAP3/CD69/APOL6/P2RY14/RSAD2/PTGS2/OAS2/IFI44/XAF1/AUTS2/CFH/CMPK2/DDX60/IFI44L/NAMPT/SELP/HERC6/IFIT1/OASL/TXNIP/C1R/GCH1/FAS/C1S/SERPING1/IFI27/MT2A
HALLMARK_SPERMATOGENESIS	HALLMARK_SPERMATOGENESIS	83	0.4215226444	1.4652152917165	0.01146788474	0.03372907278	0.01846222931	1454	tags=24%, list=10%,	GAD1/KIF2C/MLF1/TTK/CDK1/PCSK1N/NEK2/NCAPH/CCNB2/BUB1/IL13RA2/NEFH/CDKN3/CLGN/RPL39L/EZH2/RFC4/CNIH2/CFTR/DMC1

			55174		74475	66104	47762		signal=22%	
HALLMARK_EPITHELIAL_MESENCHYMAL_TRANSITION	HALLMARK_EPITHELIAL_MESENCHYMAL_TRANSITION	192	0.361867602855017	1.4133123565095	0.0129108271823305	0.0358634088398068	0.01963049747021	2157	tags=27%, list=14%, signal=23%	DKK1/LAMC2/SLC6A8/CXCL1/MMP1/PTHLH/CTHRC1/LAMA1/APLP1/FBLN1/ENO2/CXCL6/QSOX1/OXTR/PFN2/COL11A1/PMEPA1/SPP1/CDH6/SERPINE2/CAPG/GPX7/MATN3/MMP14/TNFRSF11B/FBN2/TPM2/CXCL8/CD44/EMP3/MCM7/GLIPR1/SERPINH1/TGFB1/PLAUR/DAB2/ADAM12/COMP/NTM/LGALS1/MAGEE1/LAMC1/ITGAV/RGS4/PDLIM4/TPM4/COL8A2/PLOD2/ITGA2/IGFBP3/FLNA

Table S5. Correlation analysis of prognostic genes and immune checkpoints.

	checkpoint	gene	r	p
1	SLC26A6	CD27	-0.005518841	0.91654547504406
2	TYRO3	CD27	0.263352418550811	3.57370028004346e-07
3	PDK4	CD27	-0.255106772	8.41861630569035e-07
4	SLC26A6	CD44	0.0649433480221028	0.217064007797412
5	TYRO3	CD44	0.212224504038435	4.58174719225868e-05
6	PDK4	CD44	-0.138059338	0.00844014348806944
7	SLC26A6	CD70	0.0816090444183477	0.120641099228529
8	TYRO3	CD70	0.300908548900571	4.93279717086106e-09
9	PDK4	CD70	-0.327777122	1.54213340392969e-10
10	SLC26A6	CD80	0.0355979926409862	0.498972108940596
11	TYRO3	CD80	0.397539894772496	3.40914824611255e-15
12	PDK4	CD80	-0.207406709	6.85172890876561e-05
13	SLC26A6	CD86	0.00648798715097058	0.901958454305715
14	TYRO3	CD86	0.374282673629735	1.62787150422389e-13
15	PDK4	CD86	-0.239999077	3.75340960468096e-06
16	SLC26A6	CTLA4	0.157911598893469	0.00255125128583049
17	TYRO3	CTLA4	0.330909838595776	1.00633703458066e-10
18	PDK4	CTLA4	-0.346282361	1.15367875290727e-11
19	SLC26A6	HHLA2	0.24718601395817	1.8655721933777e-06
20	TYRO3	HHLA2	0.318831989973414	5.08060978063506e-10
21	PDK4	HHLA2	-0.202989761	9.82965360015074e-05
22	SLC26A6	ICOS	0.0854706848171356	0.103993265592392
23	TYRO3	ICOS	0.264953013914866	3.01581625882253e-07
24	PDK4	ICOS	-0.294497706	1.07214687495684e-08
25	SLC26A6	IDO2	-0.10512074	0.0453428623158632
26	TYRO3	IDO2	-0.217039421	3.03645911802237e-05
27	PDK4	IDO2	0.260596279288809	4.77438779907504e-07
28	SLC26A6	KIR3DL1	-0.121915433	0.020154293922675
29	TYRO3	KIR3DL1	-0.006971592	0.894691518234035
30	PDK4	KIR3DL1	0.190286722864052	0.000266027384968637
31	SLC26A6	LAG3	0.0498559702477332	0.343538496124753
32	TYRO3	LAG3	0.193953710779427	0.000200858371022949
33	PDK4	LAG3	-0.230099383	9.4924260546221e-06
34	SLC26A6	LAIR1	0.0994021046758365	0.0584882089597017
35	TYRO3	LAIR1	0.383103837398162	3.89438131905722e-14
36	PDK4	LAIR1	-0.233007826	7.25794289429905e-06
37	SLC26A6	LGALS9	0.0454831456840498	0.387572100323817
38	TYRO3	LGALS9	0.510410483539565	1.79050460398271e-25

39	PDK4	LGALS9	-0.401552992	1.69544824986643e-15
40	SLC26A6	NRP1	0.00220071239659386	0.966670380254964
41	TYRO3	NRP1	0.350222103361229	6.49500174683388e-12
42	PDK4	NRP1	0.0362001132970495	0.491735328840784
43	SLC26A6	TIGIT	0.0423122833971654	0.421548969129256
44	TYRO3	TIGIT	0.263157485407611	3.6480644139149e-07
45	PDK4	TIGIT	-0.25819384	6.12930171401659e-07
46	SLC26A6	TNFRSF14	0.227912720127688	1.15883971908142e-05
47	TYRO3	TNFRSF14	0.225397405206546	1.45427188045977e-05
48	PDK4	TNFRSF14	-0.209197884	5.90594494242464e-05
49	SLC26A6	TNFRSF18	0.179392430773646	0.000594755587857268
50	TYRO3	TNFRSF18	0.429465311183041	1.00593076926482e-17
51	PDK4	TNFRSF18	-0.483058127	1.27444021711643e-22
52	SLC26A6	TNFRSF25	0.105572227872579	0.0444201594414819
53	TYRO3	TNFRSF25	0.295288087101246	9.75287797243889e-09
54	PDK4	TNFRSF25	-0.111650028	0.0334587423198807
55	SLC26A6	TNFRSF8	-0.016452913	0.754728881687575
56	TYRO3	TNFRSF8	0.389016656125568	1.4569509032222e-14
57	PDK4	TNFRSF8	-0.275089871	1.00290844067867e-07
58	SLC26A6	TNFRSF9	0.120713821766841	0.0214259707489257
59	TYRO3	TNFRSF9	0.2987929445725	6.38665088317094e-09
60	PDK4	TNFRSF9	-0.181786115	0.000500312539622897
61	SLC26A6	TNFSF15	0.0664165066475463	0.206792537715776
62	TYRO3	TNFSF15	0.528611017310164	1.6117940647738e-27
63	PDK4	TNFSF15	-0.415912064	1.28739236851127e-16
64	SLC26A6	TNFSF4	0.227740867871456	1.17705584992064e-05
65	TYRO3	TNFSF4	0.426821295229131	1.66912026083949e-17
66	PDK4	TNFSF4	-0.145562624	0.005459552558813
67	SLC26A6	TNFSF9	0.166998247851167	0.0014069442462353
68	TYRO3	TNFSF9	0.422397459891059	3.85616160931202e-17
69	PDK4	TNFSF9	-0.260963755	4.59438427199085e-07

SUPPLEMENTARY FIGURE LEGENDS

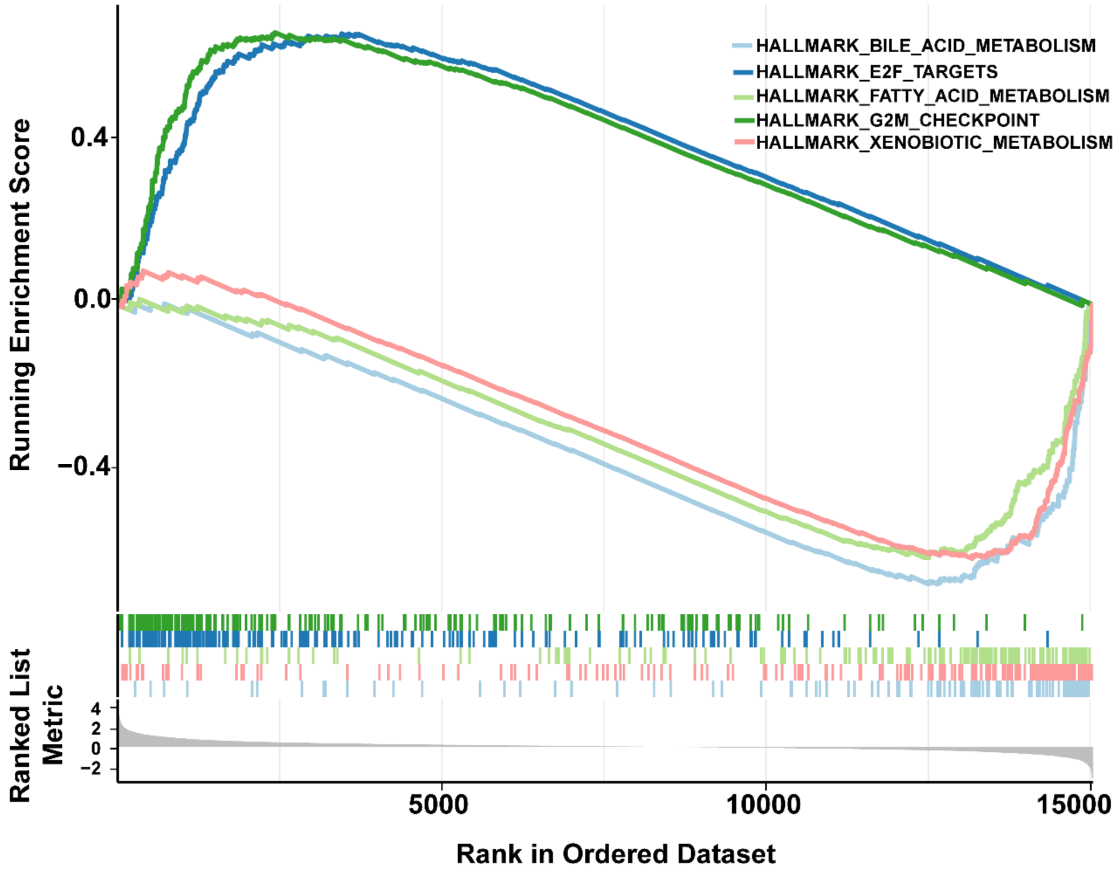

Fig. S1 Enrichment pathway analysis of Hallmark.

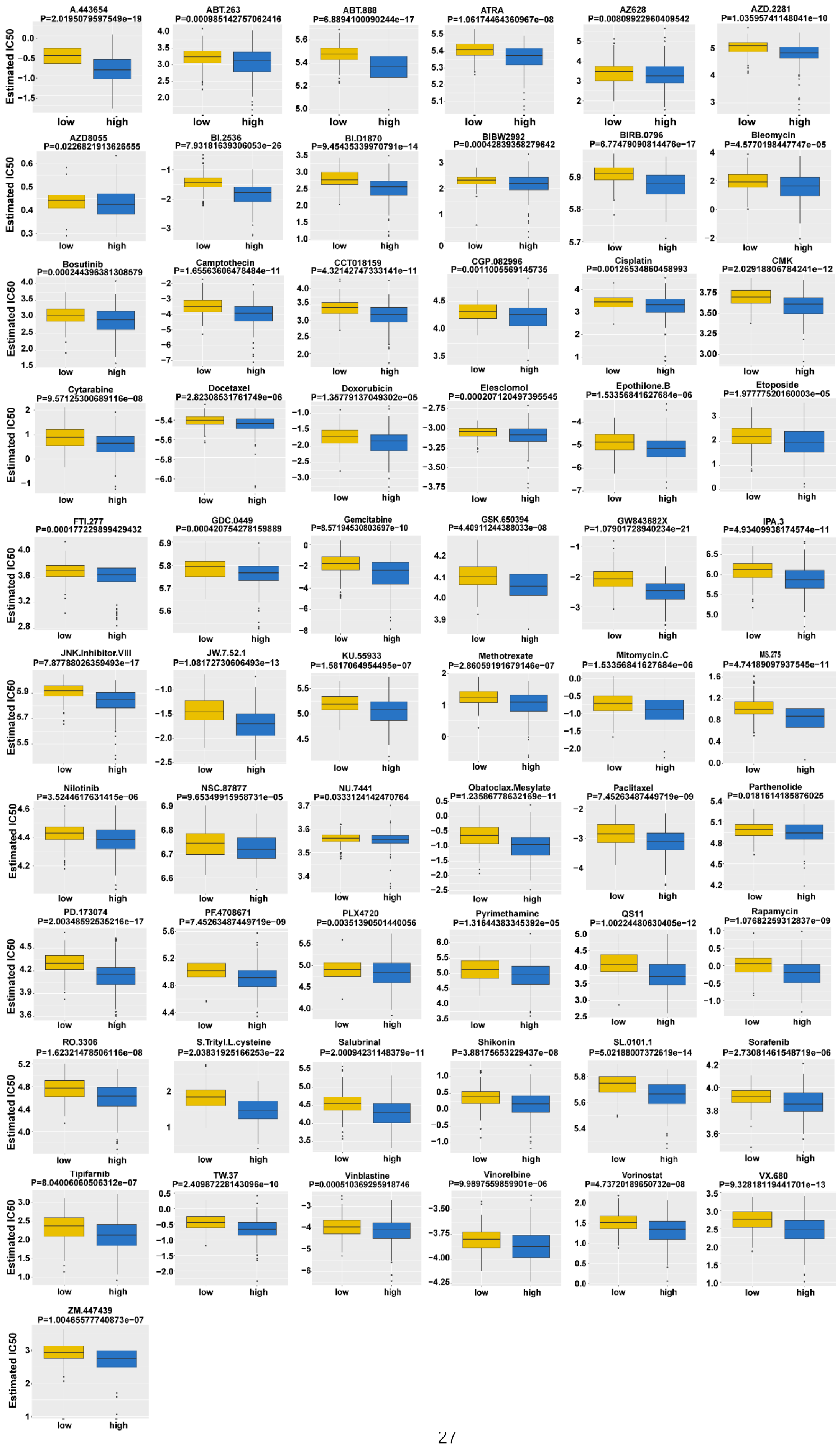
Estimated IC50
low
high
A.443654
P=2.0195079597549e−19
ABT.263
P=0.000985142757062416
ABT.888
P=6.8894100090244e−17
ATRA
P=1.06174464360967e−08
AZ628
P=0.00809922960409542
AZD.2281
P=1.03595741148041e−10
AZD8055
P=0.0226821913626555
BI.2536
P=7.93181639306053e−26
BI.D1870
P=9.45435339970791e−14
BIBW2992
P=0.00042839358279642
BIRB.0796
P=6.77479090814476e−17
Bleomycin
P=4.5770198447747e−05
Bosutinib
P=0.000244396381308579
Camptothecin
P=1.65563606478484e−11
CCT018159
P=4.32142747333141e−11
CGP.082996
P=0.0011005569145735
Cisplatin
P=0.00126534860458993
CMK
P=2.02918806784241e−12
Cytarabine
P=9.57125300689116e−08
Docetaxel
P=2.82308531761749e−06
Doxorubicin
P=1.35779137049302e−05
Elesclomol
P=0.000207120497395545
Epothilone.B
P=1.53356841627684e−06
Etoposide
P=1.97777520160003e−05
FTI.277
P=0.000177229899429432
GDC.0449
P=0.000420754278159889
Gemcitabine
P=8.57194530803697e−10
GSK.650394
P=4.40911244388033e−08
GW843682X
P=1.07901728940234e−21
IPA.3
P=4.93409938174574e−11
JNK.Inhibitor.VIII
P=7.87788026359493e−17
JW.7.52.1
P=1.08172730606493e−13
KU.55933
P=1.5817064954495e−07
Methotrexate
P=2.86059191679146e−07
Mitomycin.C
P=1.53356841627684e−06
MS.275
P=4.74189097937545e−11
Nilotinib
P=3.52446176314150e−06
NSC.87877
P=9.65349915958731e−05
NU.7441
P=0.0333124142470764
Obatoclax.Mesylate
P=1.23586778632169e−11
Paclitaxel
P=7.45263487449719e−09
Parthenolide
P=0.0181614185876025
PD.173074
P=2.00348592535216e−17
PF.4708671
P=7.45263487449719e−09
PLX4720
P=0.00351390501440056
Pyrimethamine
P=1.31644383345392e−05
QS11
P=1.00224480630405e−12
Rapamycin
P=1.07682259312837e−09
RO.3306
P=1.62321478506116e−08
S.Trityl.L.cysteine
P=2.03831925166253e−22
Salubrinal
P=2.00094231148379e−11
Shikonin
P=3.88175653229437e−08
SL.0101.1
P=5.02188007372619e−14
Sorafenib
P=2.73081461548719e−06
Tipifarnib
P=8.04006060506312e−07
TW.37
P=2.40987228143096e−10
Vinblastine
P=0.000510369295918746
Vinorelbine
P=9.9897559859901e−06
Vorinostat
P=4.73720189650732e−08
VX.680
P=9.32818119441701e−13
ZM.447439
P=1.00465577740873e−07

34

Fig. S2. IC50 values of 61 drugs differ significantly between distinct risk groups ($P < 0.05$).

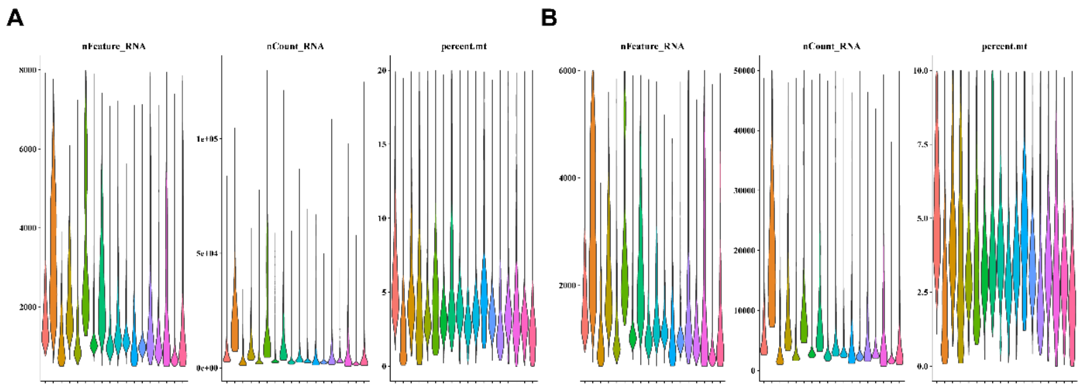

Fig. S3. Violin plots of nFeature_RNA, nCount_RNA and percent_mt before and after quality control of single-cell data. A. Violin plot before quality control. B. Violin plot after quality control.
